# The acute phase reactant orosomucoid-2 directly promotes rheumatoid inflammation

**DOI:** 10.1038/s12276-024-01188-0

**Published:** 2024-04-01

**Authors:** Ki-Myo Kim, Kang-Gu Lee, Saseong Lee, Bong-Ki Hong, Heejae Yun, Yune-Jung Park, Seung-Ah Yoo, Wan-Uk Kim

**Affiliations:** 1 Center for Integrative Rheumatoid Transcriptomics and Dynamics, The Catholic University of Korea, Seoul, South Korea; 2 Department of Biomedicine & Health Sciences, College of Medicine, The Catholic University of Korea, Seoul, South Korea; 3 Division of Rheumatology, Department of Internal Medicine, St. Vincent’s Hospital, The Catholic University of Korea, Suwon, South Korea; 4 Department of Internal Medicine, The Catholic University of Korea, Seoul, South Korea

**Keywords:** Autoimmunity, Rheumatoid arthritis

## Abstract

Acute phase proteins involved in chronic inflammatory diseases have not been systematically analyzed. Here, global proteome profiling of serum and urine revealed that orosomucoid-2 (ORM2), an acute phase reactant, was differentially expressed in rheumatoid arthritis (RA) patients and showed the highest fold change. Therefore, we questioned the extent to which ORM2, which is produced mainly in the liver, actively participates in rheumatoid inflammation. Surprisingly, ORM2 expression was upregulated in the synovial fluids and synovial membranes of RA patients. The major cell types producing ORM2 were synovial macrophages and fibroblast-like synoviocytes (FLSs) from RA patients. Recombinant ORM2 robustly increased IL-6, TNF-α, CXCL8 (IL-8), and CCL2 production by RA macrophages and FLSs via the NF-κB and p38 MAPK pathways. Interestingly, glycophorin C, a membrane protein for determining erythrocyte shape, was the receptor for ORM2. Intra-articular injection of ORM2 increased the severity of arthritis in mice and accelerated the infiltration of macrophages into the affected joints. Moreover, circulating ORM2 levels correlated with RA activity and radiographic progression. In conclusion, the acute phase protein ORM2 can directly increase the production of proinflammatory mediators and promote chronic arthritis in mice, suggesting that ORM2 could be a new therapeutic target for RA.

## Unveiling ORM2: the new player in rheumatoid arthritis progression

This research aimed to explore the function of Orosomucoid-2, a protein produced during the body’s initial response to injury, in rheumatoid arthritis, a chronic inflammatory disorder. It was found that ORM2 was produced in large quantities by synovial fibroblasts in RA patients. When these cells were treated with ORM2, they produced inflammation-causing substances, indicating ORM2 could contribute to RA inflammation. The study also identified Glycophorin C as a receptor for ORM2 on these cells. In mice, ORM2 was found to worsen arthritis and increase the presence of macrophages. In RA patients, the levels of ORM2 in the blood correlated with disease activity and progression. These findings suggest that ORM2 could be a potential diagnostic marker and treatment target for RA. More research is needed to further understand ORM2’s role in other chronic inflammatory diseases.

This summary was initially drafted using artificial intelligence, then revised and fact-checked by the author.

## Introduction

The acute phase reaction is a systemic reaction of organisms to disruptions in homeostasis such as infection, tissue injury, or inflammation. As a result, acute phase reactants are secreted in large amounts, predominantly in the liver^1^. These reactants include serum amyloid A (SAA), C-reactive protein (CRP), fibrinogen, hemopexin, orosomucoid (ORM), tumor necrosis factor-α (TNF-α), interleukin (IL)-1, IL-6, and cortisol^2–4^. Since most of these acute phase reactants can be rapidly secreted into the blood in response to infection or inflammation, they have been utilized as serum diagnostic markers to predict the activity and outcomes of chronic inflammatory diseases.

In recent years, evidence has emerged that some of these acute phase reactants are more than simple diagnostic markers. Studies have shown that these compounds can directly function as immune modulators. For example, the administration of CRP can reduce disease progression in lupus animal models^5^. In contrast, CRP can promote the activation of synoviocytes in RA patients via the NF-κB pathway^6^. SAA also exacerbates the severity of T_H_17-dependent immune diseases, such as experimental autoimmune encephalitis^7^. Our group reported that SAA is directly involved in the destruction of bone and cartilage possibly by increasing synoviocyte hyperplasia and angiogenesis in addition to its role as a diagnostic marker in RA^8^. Hemopexin, another acute phase reactant, has been demonstrated to be capable of negatively regulating the Th17 response and autoimmune disease^9^. Together, these reports raise the possibility that acute phase proteins can play active roles in the pathogenesis of inflammatory and autoimmune diseases. However, the involvement of acute phase proteins in chronic inflammatory diseases have not been globally or systematically analyzed.

Rheumatoid arthritis (RA) is a chronic inflammatory autoimmune disease characterized by tumor-like expansion of the synovium, which consists of proliferating synoviocytes, neovascularization, and infiltrating leukocytes^10,11^. In particular, synoviocytes play a major role in the initiation and perpetuation of rheumatoid inflammation^12^. There are two types of synoviocytes: macrophage-like synoviocytes and fibroblast-like synoviocytes (FLSs). These cells can proliferate abnormally and resist apoptotic cell death, ultimately constructing a major cell population in the RA synovium^10,11^. They can also produce tissue-degrading matrix metalloproteinases, leading to the destruction of cartilage and bone^10,11^. Moreover, synoviocytes can secrete a variety of proinflammatory cytokines and chemokines, including IL-6, CXCL8 (IL-8), and CCL2, which can recruit other monocytes and neutrophils to inflamed sites, further exacerbating chronic inflammation in RA^10,11^.

In the present study, global proteome profiling of serum and urine revealed that orosomucoid-2 (ORM2), an acute phase reactant, was differentially expressed in rheumatoid arthritis (RA) patients and exhibited the highest fold change. Therefore, we questioned the extent to which ORM2, which is produced mainly in the liver, actively participates in rheumatoid inflammation. Surprisingly, ORM2 expression was upregulated at extrahepatic sites, including the synovial fluids and synovial membranes of RA patients. ORM2 interacts with its receptor glycophorin C (GYPC) on synovial macrophages and fibroblasts and directly upregulates the production of proinflammatory cytokines and chemokines via the NF-κB and p38 MAP kinase pathways. This study also functionally validated the proinflammatory role of ORM2 in vivo and demonstrated positive correlations between serum ORM2 levels and both RA activity and progression. Overall, ORM2, an acute phase protein, can directly promote chronic inflammation, suggesting that ORM2 could be a novel diagnostic and therapeutic target for RA.

## Materials and methods

### Integrated analysis of proteome data

We previously identified 134 differentially expressed proteins (DEPs, 133 genes) in urine samples from rheumatoid arthritis (RA) patients compared with osteoarthritis (OA) patients^13^ through an integrated proteome analysis. Total proteins were extracted from urine samples collected from 20 RA patients and 19 OA patients via SDS‒PAGE fractionation and analyzed by liquid chromatography tandem-mass spectrometry (LC‒MS/MS). We also searched for previously published serum proteome data and identified 136 DEPs (106 genes) in the sera of RA patients (*n* = 35) compared to healthy individuals (*n* = 60) from a study in which TMT quantitation (Thermo Fisher Scientific, Waltham, MA, USA) was used for proteome analysis^14^. A total of 46 acute phase response genes (GO:0006953, http://amigo.geneontology.org/amigo/term) were found. The three lists of urine DEPs, serum DEPs, and acute phase response genes were compared to identify the shared DEPs related to the “acute phase response” between the urine and serum of RA patients. The ‘ComplexHeatmap’ package^15^ was used to construct a heatmap of the shared DEPs related to the acute phase response among the following three categories: (1) urine DEPs, (2) serum DEPs, and (3) acute phase response genes.

### Patients and assessment of RA

A total of 176 patients with RA were consecutively recruited from our outpatient clinics. All of the RA patients fulfilled the 2010 American College of Rheumatology criteria for RA^16^. Disease activity and severity of RA were evaluated at the time of blood sampling based on the disease activity score obtained from the 28-joint assessment using the erythrocyte sedimentation rate (DAS28_ESR_)^17^ and the radiographic damage of hands and feet visualized by X-ray imaging, respectively. In 90 of the 176 RA patients, hand and foot X-rays were serially monitored for 2 years. The radiographic damage score was the sum of the erosion and joint space narrowing scores according to van der Heijde’s modification of the Sharp method^18^. The maximum total score was 448. Progression scores were calculated by subtracting the baseline total score from the follow-up score after 2 years. Radiographic progression was defined as a progression score ≥4^19^. Two board-certified physicians who were blinded to each patient’s identity and clinical status assessed the patients. The interobserver variability described by the interclass correlation coefficient was 0.91. Age- and sex-matched patients with OA (*n* = 109) were used as non-RA controls. The baseline demographic characteristics of the RA patients according to radiographic progression are summarized in Supplementary Table 1.

### Transfection of NF-κB p65, p38, and GYPC siRNAs

Scrambled control siRNA and NF-κB p65, p38 MAP kinase, and GYPC siRNAs were purchased from Santa Cruz Biotechnology. RA-FLSs were transfected with 50 nM siRNAs using Lipofectamine 3000 reagent (Thermo Fisher Scientific) in Opti-MEM (Thermo Fisher Scientific) following the manufacturer’s instructions. After 6 h of transfection, the culture medium was replaced with DMEM containing 10% FBS. At 24 h after transfection, the cells were harvested for further experiments. The efficiency of the *NF-κB p65, p38*, and *GYPC* siRNAs was determined by qRT‒PCR and/or Western blotting.

### Immunocytochemistry for NF-κB p65

To determine the intracellular localization of NF-κB, immunofluorescence staining of p65 (ab7970; Abcam, Cambridge, England) in RA-FLSs was performed. In brief, RA-FLSs (1 × 10^5^ cells per well) were seeded into an eight-well chamber (Thermo Fisher Scientific) and grown to 80% confluence for 24 h. Recombinant human ORM2 (1 μg/mL; Prospec-Tany TechnoGene Ltd., Rehovot, Israel) was added to each well and incubated for 1 h. Cells were fixed with formaldehyde for 10 min and permeabilized with 0.1% Triton X-100 for 3 min at room temperature. After blocking with 10% normal donkey serum at room temperature for 1 h, the cells were stained with an anti-p65 Ab (ab7970, 1:100; Abcam) and Alexa 488-conjugated donkey anti-IgG (a21206, 1:1000; Thermo Fisher Scientific). After washing with PBS three times, the nuclei were stained with DAPI (1:500; BD Biosciences, San Jose, CA, USA). The stained cells were visualized with a confocal microscope (Zeiss, LSM800, Gottingen, Germany).

### Prediction of ORM2-protein interactions

Protein interaction data of ORM2 were obtained from the following five interactome databases: the Biological General Repository for Interaction Datasets (BioGRID)^20^, the Human Protein Reference Database (HPRD)^21^, the Human Transcriptional Regulation Interactions database (HTRIdb)^22^, the IntAct Molecular Interaction database (IntAct)^23^, and the Search Tool for the Retrieval of Interacting Genes/Proteins (STRING)^24^.

### Proximity ligation assay

RA-FLSs (4 × 10^3^ cells per well) were seeded in eight-well chambers (Thermo Fisher Scientific) and grown to 80% confluence for 24 h. Recombinant ORM2 (1 μg/mL; Prospec-Tany Technogene) was added to each well and incubated at 37 °C for 1 h. The cells were then fixed with formaldehyde, permeabilized with 0.1% Triton X-100 for 3 min at room temperature, and incubated with an anti-ORM2 Ab (bs-7565R, 1:100; Bioss, Woburn, MA, USA) and/or an anti-GYPC Ab (sc-59183, 1:100; Santa Cruz Biotechnology) for 1 h at 37 °C. These cells were then stained with a Duolink In Situ Orange Starter Kit Mouse/Rabbit (Sigma‒Aldrich, St. Louis, MO, USA) according to the manufacturer’s instructions. Approximately 50–100 cells per area (at a magnification of 100×) were observed, and more than 5 randomly selected areas were subjected to analysis.

### Solid-phase ELISA to determine the molecular interaction between ORM2 and GYPC

Recombinant human GYPC protein (0, 0.5, and 5 μg, Prospec-Tany Technogene) diluted in phosphate-buffered saline (PBS) was coated onto high-protein-binding 96-well plates (Corning, Inc., Corning, NY, USA) overnight at room temperature. After three washes with PBS containing 0.05% Tween 20 (PBS-T), blocking buffer containing 1% bovine serum albumin (BSA) in PBS-T was added to the 96-well plates, and the plates were incubated for 2 h at room temperature. Recombinant human ORM2 (Prospec-Tany Technogene) or BSA (Bovogen, Keilor East, VIC, AUS) at concentrations of 0, 5, 25, and 125 μg was then added to the plate and incubated overnight at 4 °C. After three washes with PBS-T, rabbit anti-ORM2 Ab (bs-7565r, Bioss) was added to the plates, and the reactions were allowed to proceed for 2 h at room temperature. After washing three times with PBS-T, goat anti-rabbit IgG Ab conjugated with HRP (21040 s, Thermo Fisher Scientific) was added as the secondary Ab, and the reaction was again allowed to proceed for 1 h at room temperature. After three additional washes with PBS-T, TMB solution was added to induce a color reaction, which was then stopped with H_2_SO_4_ (sulfuric acid) solution. Finally, the optical densities were read at a wavelength of 450 nm.

### Induction of ORM2- accelerated arthritis in mice

Eight-week-old C57BL/6 N mice were used for the in vivo experiment. Following previously published protocols^25^, 10 μL of methylated bovine serum albumin (mBSA; Sigma‒Aldrich) at a concentration of 20 mg/mL was intra-articularly injected into both knee joints of each mouse on Day 0. On the same day (Day 0), recombinant mouse ORM2 (4 μg; Mybiosource, San Diego, CA, USA) was administered intra-articularly into a unilateral knee joint. The opposite knee joint was injected with the vehicle alone. Recombinant IL-1β (250 ng; R&D Systems) was then subcutaneously injected into the footpad ipsilateral to the ORM2 injection site twice on Days 1 and 2. A separate control cohort of mice was used in which vehicle was injected instead of ORM2 but otherwise were subjected to the same procedure as the group with ORM2-accelerated arthritis underwent. These mice were sacrificed on Day 7. Histological analysis was subsequently performed on the mouse joints.

### Histological assessment of arthritis

Knee joints were exposed by removing the overlying skin and subsequently excised. The limbs were fixed in 10% formalin solution (Sigma‒Aldrich) at room temperature overnight and decalcified in 5% formic acid for 10–14 days. Tissues were cut into 4 μm sections and stained with hematoxylin and eosin (Sigma‒Aldrich). The degrees of inflammation, synovial hyperplasia, and bone destruction in the joints were determined using a standard scoring protocol^26^. Each of the three parameters was scored based on a scale of 0 to 3 (0 = absent, 1 = weak, 2 = moderate, and 3 = severe).

### Statistical analysis

All of the statistical analyses were carried out using GraphPad Prism v.8.4.3. The normality of the data distribution was verified using the Shapiro‒Wilk test or Kolmogorov‒Smirnov test, and the homogeneity of variance was tested using the F test or Brown–Forsythe test. Differences between two groups were analyzed by unpaired two-tailed *t* tests, Welch’s *t* tests or Mann‒Whitney U tests. Comparisons among multiple groups were performed by one-way ANOVA with Tukey’s multiple comparisons test, Brown-Forsythe and Welch ANOVA with Dunnett T3 multiple-comparison test, or Kruskal‒Wallis test with Dunn’s multiple comparisons test or Mann‒Whitney post hoc test. For time-course or dose‒response data, two-way ANOVA with Tukey’s multiple comparisons test, Friedman’s test with Dunn’s multiple comparisons test or the Mann‒Whitney post hoc test was used to determine statistical significance. Correlation analysis was performed using the Spearman test. The data are shown as the mean ± standard deviation (SD) or standard error of the mean (SEM). *P* < 0.05 were considered to indicate statistical significance.

### Study approval

This study was performed with the approval of the institutional review board (KC14TASI0898) at Seoul St. Mary’s Hospital. All animal procedures were performed in accordance with the Laboratory Animals Welfare Act, the Guide for the Care and Use of Laboratory Animals and the Guidelines and Policies for Rodent Experiment provided by the Institutional Animal Care and Use Committee of the Catholic University of Korea (approval number, CUMS-2015-0112-02 and CUMS-2018-0062-03).

## Results

### ORM2 expression is upregulated in RA patients by proinflammatory stimuli

**Fig. 1:**
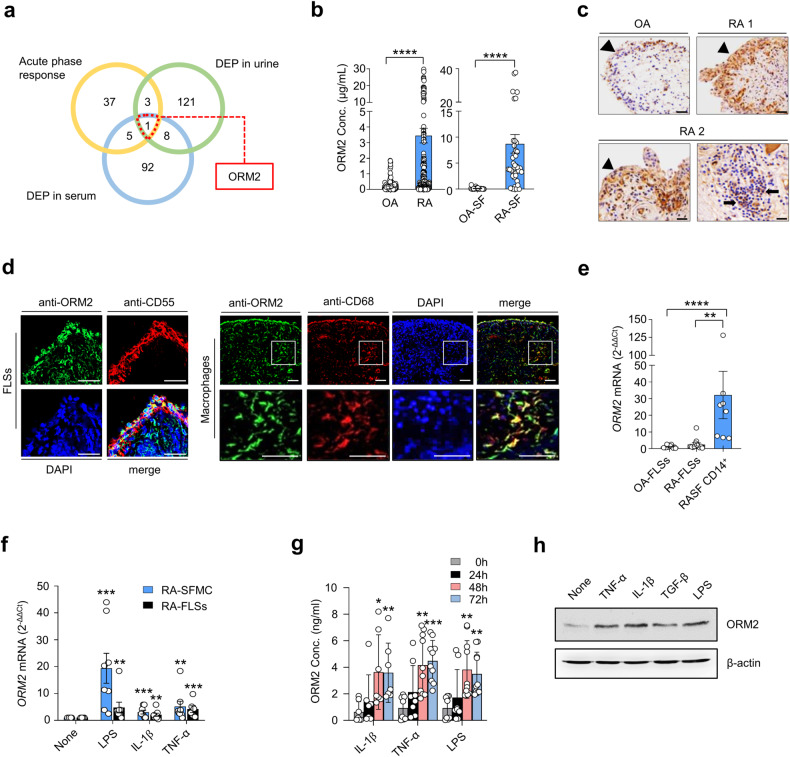
Expression of ORM2 in the synovia, synovial fluids, and synoviocytes of RA patients. **a** Venn diagram depicting the number of common and distinct acute phase response proteins, differentially expressed proteins (DEPs) in RA patient urine, and DEPs in RA patient serum. **b** ORM2 concentrations in the sera of RA patients (*n* = 179, left panel), the sera of osteoarthritis (OA) patients (*n* = 109, left panel), the synovial fluids of RA patients (RA-SF; *n* = 40, right panel), and the synovial fluids of OA patients (OA-SF; *n* = 25, right panel) as determined by ELISA. The bar graphs represent the mean ± SD. ****P < 0.0001 according to the Mann‒Whitney U test. **c** Immunohistochemical staining for ORM2 in the synovial tissues of RA patients (RA1 and RA2) and an OA patient using anti-ORM2 antibodies (Abs). Arrowheads and arrows indicate the lining layer and sublining leukocytes, respectively. Scale bars: 50 μm. **d** Double immunofluorescence staining of RA synovial tissue using Abs against ORM2, CD55, and CD68. Scale bars: 50 μm. The rectangular area in the top panel is magnified to the bottom panel. Scale bars: 50 μm. See Supplementary Fig. 2 for the immunofluorescence staining of the synovium from three other RA patients. **e** qRT‒PCR assays for *ORM2* expression in cultured fibroblast-like synoviocytes from OA patients (OA-FLSs, *n* = 12), cultured FLSs from RA patients (RA-FLSs, *n* = 12), and CD14^+^ macrophages/monocytes (*n* = 8) isolated from the synovial fluids of RA patients as determined by qRT‒PCR. *ORM2* mRNA expression levels were first normalized to those of *GAPDH* (internal control) and subsequently further normalized to the mean mRNA expression level in OA-FLSs. **f** Induction of ORM2 in RA mononuclear cells and RA-FLSs by LPS and proinflammatory cytokines. Mononuclear cells (5 × 10^5^) freshly isolated from the synovial fluid of RA patients (RA-SFMCs) and RA-FLSs (2 × 10^5^) were stimulated with LPS (1 μg/mL), TNF-α (10 ng/mL), or IL-1β (1 ng/mL) for 24 h. *ORM2* mRNA expression levels, which were determined by qRT‒PCR, were first normalized to the expression of *GAPDH* and subsequently further normalized to the mean mRNA expression in unstimulated cells. **g** ORM2 secretion by CD14^+^ cells. CD14^+^ cells were isolated from RA synovial fluids and then stimulated with IL-1β (1 ng/mL), TNF-α (10 ng/mL), and LPS (1 μg/mL) for the indicated times. ORM2 levels in culture supernatants were measured via ELISA. **h** Western blot analysis of ORM2 expression in RA-FLSs. RA-FLSs were stimulated with TNF-α (10 ng/mL), IL-1β (1 ng/mL), TGF-β (10 ng/mL), and LPS (1 μg/mL) for 48 h; a representative of more than three experiments is shown. The data are presented as the mean ± SEM of more than three independent experiments. *P < 0.05, **P < 0.01, ***P < 0.001, and ****P < 0.0001 according to the Kruskal–Wallis test (E: *P* = 0.0001, RA-SFMC in (**f**): *P* = 0.0001; RA-FLSs in (**f**): *P* = 0.0027; IL-1β in (**g**): *P* = 0.0025; TNF-α in (**g**): *P* = 0.0002; and LPS in (**g**): *P* = 0.0004) with Dunn’s multiple comparisons test for (**e**) and (**g**) or with post hoc pairwise comparisons test using a Mann–Whitney U test for (**f**).

A global analysis to determine which acute phase reactants are involved in rheumatoid inflammation has not yet been performed. To address this knowledge gap, we conducted a comparative analysis of DEPs in RA sera (106 genes), DEPs in RA urine (133 genes), and 46 genes defined as acute phase reactants by Gene Ontology (GO) (Fig. 1a). We identified 9 acute phase reactants upregulated in the sera and/or urine samples of RA patients. Among these genes, ORM2 exhibited the highest fold change. Thus, it was selected as a potential regulator involved in RA pathology (Fig. 1 and Supplementary Fig. 1). Indeed, the ORM2 expression levels were much greater in the sera and synovial fluids of the RA patients than in those of the OA patients (mean ± SEM: 3.5 ± 0.47 μg/mL vs. 0.26 ± 0.04 μg/mL in the sera and 8.7 ± 1.8 μg/mL vs. 0.12 ± 0.04 μg/mL in the synovial fluids) (Fig. 1b). In addition, immunohistochemical staining revealed that ORM2 was more highly expressed in RA synovia than in OA synovia, particularly in the lining layer and sublining leukocytes (Fig. 1c).

The lining of the synovium in RA patients is composed of macrophage-like synoviocytes and FLSs^27^. Immunofluorescence staining revealed that ORM2-expressing cells colocalized well with CD55^+^ and CD68^+^ cells, which are markers of FLSs and macrophages, respectively, indicating that these cells were the major cells expressing ORM2 (Fig. 1d and Supplementary Fig. 2a, b). The use of only the secondary Abs without either the anti-ORM2 Ab or the anti-CD68 Ab failed to stain the ORM2- or CD68-expressing cells, respectively (Supplementary Fig. 2c). Moreover, preabsorption of the anti-ORM2 Ab with recombinant ORM2 markedly inhibited ORM2 staining in CD68^+^ cells (Supplementary Fig. 2d), indicating the specificity of the anti-ORM2 Ab. To further confirm the presence of ORM2 in the RA synovium, we isolated FLSs and macrophages from synovial membranes and synovial fluids, respectively, and measured ORM2 expression levels. As a result, freshly isolated CD14^+^ cells from RA patients had significantly higher levels of *ORM2* mRNA than FLSs from RA patients (RA-FLSs) and FLSs from OA patients (OA-FLSs) (Fig. 1e). The mean *ORM2* mRNA expression levels in RA CD14^+^ cells were 12.5-fold and 27.8-fold higher than those in RA-FLSs and OA-FLSs, respectively (Fig. 1e). The expression of *ORM2* mRNA was 2.2-fold higher in RA-FLSs than in OA-FLSs, but this difference was not statistically significant (*p* = 0.11) (Fig. 1e).

The RA synovium is heavily exposed to a variety of proinflammatory stimuli, including Toll-like receptor ligands and inflammatory cytokines. As shown in Fig. 1f, stimulation of synovial fluid mononuclear cells (SFMCs) from RA patients (RA-SFMCs) with LPS, IL-1β, or TNF-α resulted in a substantial increase in *ORM2* mRNA expression levels—by 19.4-, 3.1-, and 5.2-fold, respectively—compared to treatment with medium alone. This increase in *ORM2* mRNA expression levels was not observed in macrophages treated with IL-6 (Supplementary Fig. 3a**)**. *ORM*2 transcript levels in RA-FLSs were also significantly increased by LPS, IL-1β, TNF-α, and TGF-β stimuli—up to 4.8-, 2.1-, 4.2-, and 2.9-fold, respectively—but not by other cytokines, such as IL-6, M-CSF, or IL-10 (Fig. 1f and Supplementary Fig. 3b). ORM2 protein production was also markedly increased in the synovial fluid CD14^+^ cells of RA patients and in RA-FLSs after stimulation with proinflammatory stimuli, including LPS, IL-1β, and TNF-α, as well as the profibrotic cytokine TGF-β (Fig. 1g, h).

In summary, ORM2 expression was upregulated in the extrahepatic sites, including synovial fluids and synovial membranes, of RA patients, and it was upregulated by proinflammatory stimuli. The major cell types producing ORM2 were synovial macrophages and fibroblasts.

### ORM2 directly increases the production of IL-6, CXCL8, and CCL2 by RA-FLSs and macrophages

**Fig. 2:**
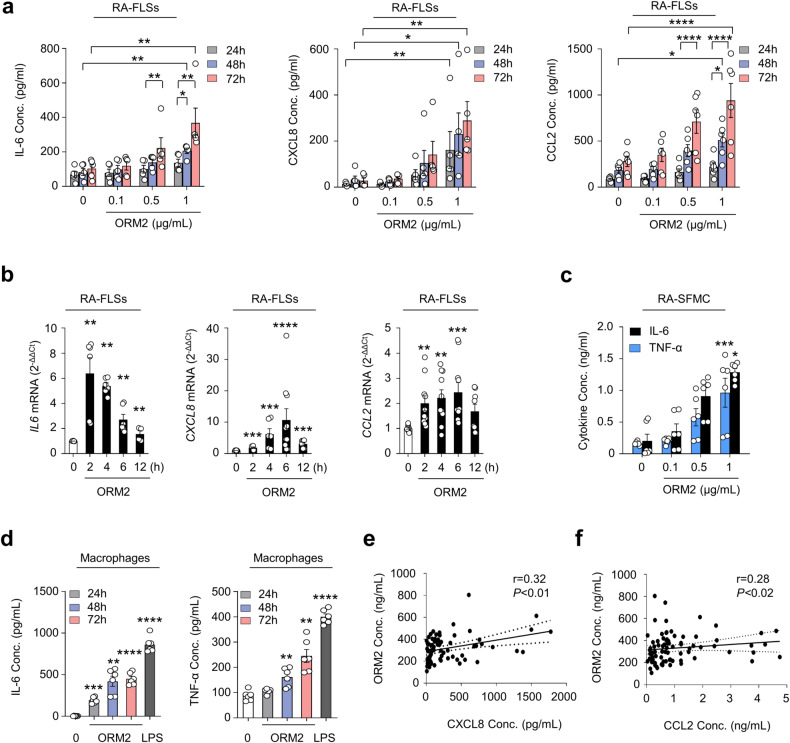
Increase in proinflammatory cytokine production after treatment with recombinant ORM2. **a** Upregulation of IL-6, CXCL8, and CCL2 expression in RA-FLSs induced by recombinant ORM2. RA-FLSs (2 × 10^4^, *n* = 5–6) were cultured in DMEM supplemented with 1% FBS and stimulated with recombinant ORM2 at various concentrations (0.1 to 1 μg/mL) in the presence of polymyxin B (30 μg/mL) for the indicated times. The IL-6 and CXCL8 concentrations (Conc.) in the culture supernatants were measured via ELISA. **b** ORM2 upregulated the mRNA expression of the *IL6*, *CXCL8*, and *CCL2* in RA-FLSs, as determined by qRT‒PCR. RA-FLSs were stimulated with recombinant ORM2 (1 μg/mL) for the indicated times. *GAPDH* mRNA was used as an internal control. **c**, **d** ORM2 increased IL-6 and TNF-α production in synovial fluid mononuclear cells from RA patients (RA-SFMCs) (**c**) and in macrophages differentiated from peripheral monocytes (**d**). RA-SFMCs (1 × 10^6^) were freshly isolated from the synovial fluids of RA patients and then stimulated with various concentrations of ORM2 (0.1–1 μg/mL) for 72 h. Peripheral monocytes were obtained from blood samples of healthy donors (*n* = 6) and differentiated into macrophages by incubating them in the presence of M-CSF (20 ng/mL) for 3 days. The resulting macrophages (1 × 10^6^) were stimulated with recombinant ORM2 (1 μg/mL) in the presence of polymyxin B (30 μg/mL) for the indicated times. IL-6 and TNF-α levels in culture supernatants were determined via ELISA. The data in (**a**–**d**) represent the mean ± SEM of more than three independent experiments. *P < 0.05, **P < 0.01, ***P < 0.001, and ****P < 0.0001 according to the Friedman test (IL-6 in a: *P* < 0.0001, CXCL8 in A: *P* < 0.0001) with post hoc pairwise comparisons using a Mann–Whitney U test; two-way ANOVA (*P* < 0.0001) with Tukey’s multiple comparisons test for CCL2 in (**a**); Kruskal–Wallis test (*IL6* in (**b**): *P* = 0.0001; *CXCL8* in (**b**): *P* < 0.0001; CCL2 in (**b**): *P* = 0.003; and TNF-α in (**c**): *P* = 0.009) with post hoc pairwise comparisons using the Mann–Whitney U test for *IL6* and *CXCL8* in (**b**) or with Dunn’s multiple comparisons for CCL2 in (**b**) and TNF-α in (**c**); and Brown-Forsythe and Welch ANOVA (IL-6 in (**c**): *P* = 0.0084, IL-6 in (**d**): *P* < 0.0001, and TNF-α in (**d**): *P* < 0.0001) with the Dunnett T3 multiple-comparison test versus untreated cells. **e**, **f** Scatter plot presenting the correlations between the ORM2 concentration and the CXCL8 (**e**) or CCL2 (**f**) level in RA synovial fluid (*n* = 81). The data were assessed by Spearman’s correlation coefficient analysis.

Next, we investigated whether ORM2, like several other acute phase reactants^5–9^, could functionally regulate inflammatory responses and contribute to RA pathogenesis. To this end, we treated RA-FLSs and macrophages with recombinant ORM2 and tested whether ORM2 could induce the production of proinflammatory cytokines. The results showed that recombinant ORM2 dramatically increased the production of IL-6, CXCL8, and CCL2 by RA-FLSs in a dose- and time-dependent manner (Fig. 2a). The effect of recombinant ORM2 persisted even when polymyxin B, a compound that blocks LPS-induced TLR4 activation, was present. In contrast, the LPS-induced increase in *IL6* and *CXCL8* expression levels was completely inhibited by polymyxin B (Supplementary Fig. 4a). After stimulation with 1 μg/mL ORM2 for 72 h, the levels of IL-6, CXCL8, and CCL2 produced by RA-FLSs increased 3.6-, 10.3-, and 3.4-fold, respectively, relative to those observed after treatment with medium alone (Fig. 2a). These increases did not seem to be due to cell proliferation since the number and viability of FLSs were not altered according to the results of trypan blue exclusion and the MTT assay, respectively, at 72 h after stimulation with 0.1 to 1 μg/mL ORM2 (Supplementary Fig. 4b). After treatment with exogenous ORM2, the *IL6*, *CXCL8*, and *CCL2* mRNA expression levels also markedly increased by 6.4-, 10.7-, and 2.4-fold, respectively, compared to those in the medium-treated control (Fig. 2b), indicating that these increases were transcriptionally regulated. Similarly, ORM2-stimulated RA-SFMCs dose-dependently increased the production of IL-6 and TNF-α (Fig. 2c). In parallel, recombinant ORM2 time-dependently increased IL-6 and TNF-α secretion from the macrophages of healthy controls, which were differentiated from peripheral monocytes (Fig. 2d). The secretion of CXCL8 and CCL2 from healthy macrophages was also robustly promoted by exogenous ORM2 (Supplementary Fig. 4c). Furthermore, in RA synovial fluid, a modest correlation was found between the ORM2 concentration and the CXCL8 and CCL2 levels (Fig. 2e, f). Taken together, these results suggest that upregulated ORM2 in RA joints can directly stimulate RA-FLSs and macrophages, known as effector cells in RA, to induce the production of proinflammatory cytokines and chemokines, thereby further amplifying inflammatory responses.

### NF-κB and p38 are major signals for ORM2-induced proinflammatory responses

**Fig. 3:**
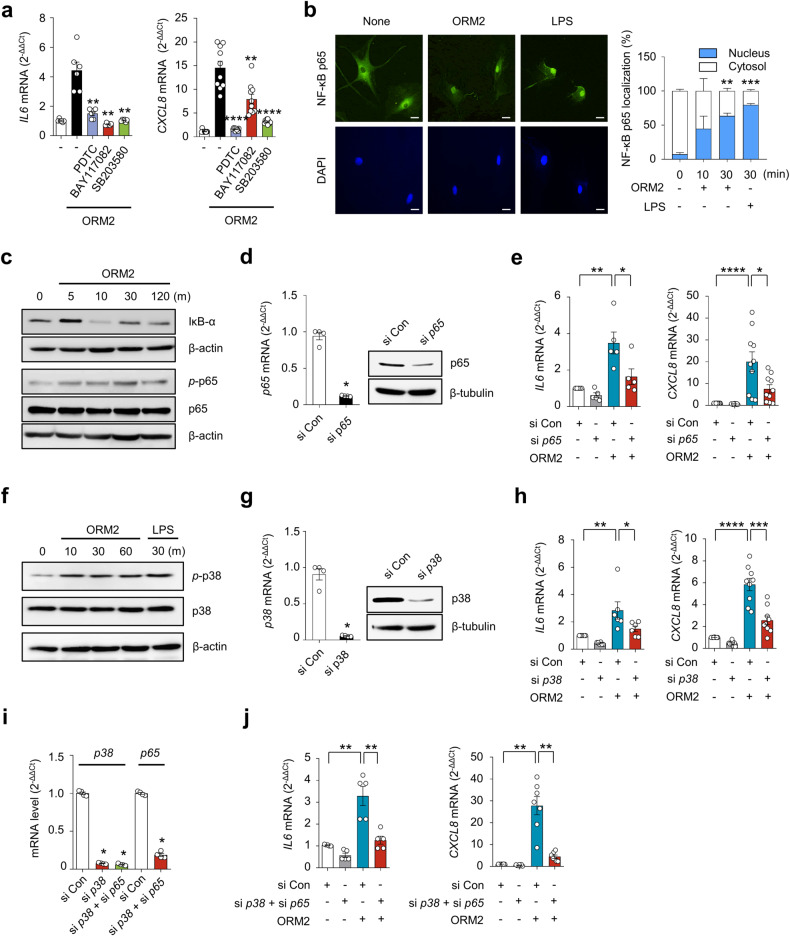
Involvement of the NF-κB and p38 pathways in ORM2-induced cytokine production. **a** Effect of NF-κB and p38 MAP kinase inhibitors on ORM2-stimulated *IL6* and *CXCL8* expression. RA-FLSs were pretreated with PDTC (10 μM), BAY 117082 (40 μM), or SB203580 (10 μM) for 1 h and then stimulated with recombinant ORM2 (1 μg/mL) for 6 h. *IL6* and *CXCL8* mRNA levels were assessed by qRT‒PCR. The data are presented as the mean ± SEM of more than three independent experiments. **P < 0.01 and ****P < 0.0001 versus ORM2 according to the Kruskal–Wallis test (*IL6*: *P* < 0.0001, *CXCL8*: *P* < 0.0001) with post hoc pairwise comparisons test using the Mann–Whitney U test. **b** Immunocytochemistry analysis of NF-κB p65 in RA-FLSs. Cells were activated with ORM2 (1 μg/mL) or LPS (100 ng/mL) for the indicated times. Representative confocal images of p65 translocation to the nucleus are presented. The extent of nuclear translocation (%) was manually counted and is presented in the bar graph. The data are presented as the mean ± SEM of more than three independent experiments. **P < 0.01 and ***P < 0.001 versus no ORM2 according to two-way ANOVA (*P* < 0.0001) with Sadik’s multiple comparisons. Scale bar: 20 μm. **c** Western blot analysis of IκB-α, NF-κB phospho-p65 (*p*-p65), and NF-κB p65 in RA-FLSs stimulated with ORM2 for the indicated times (minutes [m]). **d**, **e** Decrease in ORM2-induced *IL6* and *CXCL8* mRNA levels induced by knockdown of NF-κB *p65*. RA-FLSs were transfected with NF-κB *p65* siRNAs (si-p65, 50 nM) or control siRNAs (si Con, 50 nM) for 24 h. NF-κB *p65* expression was determined by qRT‒PCR (left in **d**) and Western blot analysis (right in **d**). *IL6* and *CXCL8* expression levels were determined by qRT‒PCR (**e**). **f** Total p38 and phospho-p38 (*p*-p38) expression levels in RA-FLSs determined by Western blotting after stimulation with ORM2 for the indicated times (minutes). **g** Downregulation of p38 expression after 24 h of transfection with *p38* siRNAs (si *p38*, 50 nM), as determined by qRT‒PCR (left) and Western blot analysis (right). **h** qRT‒PCR analysis of *IL6* and *CXCL8* expression. **i** NF-κB *p65* and *p38* expression in double-knockdown cells was analyzed via qRT‒PCR. After *p38* transcripts were knocked down for 24 h, RA-FLSs (*n* = 6) were transfected again with si-*p65* for an additional 24 h. **j** qRT‒PCR analysis of *IL6* and *CXCL8* expression. The qRT‒PCR data in (**e**), (**h**), and (**j**) were obtained for the siRNA-transfected RA-FLSs (*n* = 5) 6 h after stimulation with ORM2 (1 μg/mL). The Western blot data in (**c**), (**d**), (**f**), and (**g**) are representative of three independent experiments. The bar graphs represent the mean ± SEM. *P < 0.05, **P < 0.01, ***P < 0.001, and ****P < 0.0001 according to the Mann‒Whitney U test for (**d**) and (**g**); one-way ANOVA (*IL6* in (**e**): *P* = 0.0003; *IL6* in (**h**): *P* = 0.0004) with Tukey’s multiple comparisons test; Kruskal‒Wallis test (*CXCL8* in (**e**): *P* < 0.0001; *CXCL8* in (**h**): *P* < 0.0001; p38 in (**i**): *P* = 0.0002; and *IL6* in (**j**): *P* = 0.001) with post hoc pairwise comparisons test using a Mann‒Whitney U test; and Brown-Forsythe and Welch ANOVA (*P* < 0.0001) with Dunnett T3 multiple-comparison test for *CXCL8* in (**j**).

We further investigated which signaling pathways are involved in the regulation of cytokine and chemokine production by ORM2. A number of studies have demonstrated that NF-κB and p38 MAP kinase are major signaling molecules responsible for IL-6, CXCL8, and CCL2 production in RA-FLSs^28^. To determine how ORM2 induces IL-6 and CXCL8 production, we treated RA-FLSs with recombinant human ORM2 in the presence of chemical inhibitors specific for NF-κB and p38 MAP kinase. As expected, NF-κB inhibitors such as pyrollidine dithiocarbamate (PDTC) and BAY117082 substantially suppressed ORM2-induced increases in *IL6* and *CXCL8* mRNA expression levels (Fig. 3a); the suppressive effect of the BAY inhibitor on *IL6* upregulation was less pronounced than that on *CXCL8* upregulation, suggesting that the NF-κB pathway partially contributes to the production of *CXCL8* induced by ORM2. Additionally, the p38 MAP kinase inhibitor SB203580 strongly inhibited the upregulation of the *IL6* and *CXCL8* mRNAs in RA-FLSs stimulated with ORM2 (Fig. 3a). Similarly, in macrophages from healthy controls, the increase in *IL6* and *CCL2* expression levels induced by ORM2 was almost completely suppressed by SB203580, PDTC and BAY117082 (Supplementary Fig. 5). However, in contrast to NF-κB inhibitors, SB203580 failed to mitigate the increase in *CXCL8* expression levels in macrophages. Taken together, these results suggest that ORM2 can increase the production of proinflammatory cytokines via the NF-κB and/or p38 MAP kinase pathway in RA-FLSs and macrophages.

In support of this, recombinant human ORM2 (1 μg/mL) time-dependently increased NF-κB translocation from the cytoplasm to the nucleus in RA-FLSs and upregulated the phosphorylation of NF-κB p65 (Fig. 3b, c), while it downregulated IκB expression up to 2 h after stimulation (Fig. 3c). Moreover, NF-κB *p65* siRNA, but not control siRNA, markedly repressed the upregulation of *IL6* and *CXCL8* mRNA expression induced by ORM2 (Fig. 3d, e), indicating that NF-κB was a major signaling factor involved in this process. Moreover, as early as 10 min following stimulation with recombinant ORM2, phospho-p38 (*p*-p38) MAP kinase expression was sharply upregulated in RA-FLSs, as determined by Western blot analysis. This increase lasted for 1 h (Fig. 3f). Like NF-κB *p65* siRNA, *p38* siRNA also strongly inhibited the ORM2-induced increase in *IL6* and *CXCL8* mRNA levels (Fig. 3g, h), demonstrating that p38 MAP kinase is another major signaling molecule that mediates the promotive effect of ORM2 on IL-6 and CXCL8 expression levels in RA-FLSs. Notably, knockdown of either the NF-κB *p65* or *p38* MAP kinase transcript only partially reduced the ORM2-stimulated mRNA expression of the *IL6* and *CXCL8* (Fig. 3e, h). However, simultaneous knockdown of NF-κB *p65* and *p38* MAP kinase almost completely abolished the ORM2-induced increase in the mRNA expression levels of *IL6* and *CXCL8* (Fig. 3i, j), suggesting that both the NF-κB p65 and p38 MAP kinase signaling pathways are required for ORM2-induced IL-6 and CXCL8 production by RA-FLSs.

### Glycophorin C is a receptor for ORM2 on synovial macrophages and FLSs

**Fig. 4:**
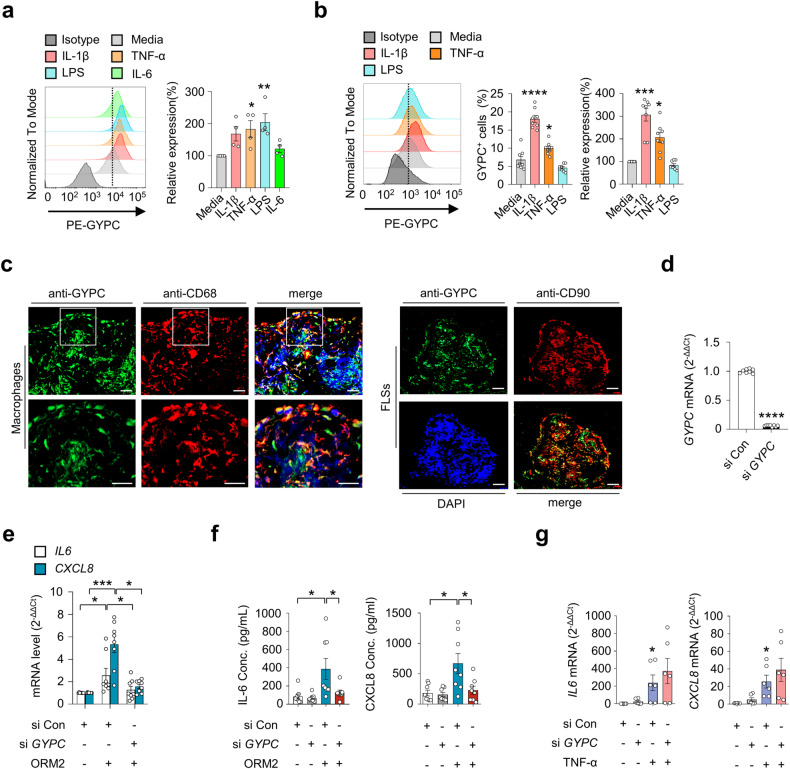
Expression and function of glycophorin C in macrophages and RA-FLSs. **a** Flow cytometry analysis of the effect of glycophorin C (GYPC) on macrophages. CD14^+^ monocytes were isolated from healthy donors (*n* = 4) and differentiated into macrophages by treatment with M-CSF (20 ng/mL) for 3 days. The cells were then cultured in the absence or presence of IL-1β (10 ng/mL), TNF-α (10 ng/mL), LPS (100 ng/mL), or IL-6 (10 ng/mL) for 24 h. A representative plot is shown in the left panel. The data are presented as the mean ± SEM. *P < 0.05, **P < 0.001 versus media alone according to one-way ANOVA (*P* = 0.01) with Dunnett’s multiple comparisons test. **b** Flow cytometry analysis of GYPC on RA-FLSs. RA-FLSs (*n* = 4) were stimulated with media alone, IL-1β (10 ng/mL), TNF-α (10 ng/mL), or LPS (100 ng/mL) for 48 h. A representative plot is shown in the left panel. The data are presented as the mean ± SEM. *P < 0.05, ***P < 0.001, and ****P < 0.0001 versus media alone according to one-way ANOVA (*P* < 0.0001) with Tukey’s multiple comparisons test for GYPC^+^ cells and the Kruskal–Wallis test (*P* < 0.0001) with Dunn’s multiple comparisons test for relative expression of GYPC. **c** Double immunofluorescence staining of an RA synovium using antibodies against GYPC, CD68, and CD90. The rectangular area in the top panel is magnified to the bottom panel. Scale bars: 50 μm. For additional immunofluorescence staining data, see Supplementary Fig. 7. **d** Knockdown of GYPC in RA-FLSs. Cells were transfected with GYPC siRNAs (si *GYPC*) for 24 h and subjected to qRT‒PCR. **e** Decrease in ORM2-induced *IL6* and *CXCL8* mRNA expression in RA-FLSs by *GYPC* knockdown. RA-FLSs were transfected with si *GYPC* or si Con for 24 h, stimulated with ORM2 (1 μg/mL) for 6 h, and then subjected to qRT‒PCR to measure *IL6* and *CXCL8* mRNA expression. **f** *GYPC* siRNA reduced IL-6 and CXCL8 secretion by RA-FLSs. The cells were transfected with si *GYPC* or si Con for 24 h and then stimulated with ORM2 for 72 h. The IL-6 and CXCL8 concentrations (Conc.) in the culture supernatants were measured via ELISA. **g** No effects of si *GYPC* on TNF-α-induced *IL6* and *CXCL8* mRNA expression was not detected via qRT‒PCR. RA*-*FLSs were transfected with si *GYPC* for 24 h and then stimulated with TNF-α (10 ng/mL) for 6 h. The bar graphs show the mean ± SEM of more than three independent experiments. *P < 0.05, **P < 0.01, ***P < 0.001, and ****P < 0.0001 according to Welch’s t test for (**d**) and the Kruskal–Wallis test (*IL6* in (**e**): *P* = 0.0159, *CXCL8* in (**e**): *P* = 0.0005; IL-6 in (**f**): *P* = 0.0022; CXCL8 in (**f**): *P* = 0.0182; *IL6* in (**g**): *P* = 0.0035; and *CXCL8* in (**g**): *P* = 0.0023) with Dunn’s multiple comparisons test for (**e**) and (**g**) or with post hoc pairwise comparisons test using a Mann–Whitney U test for (**f**).

Given the ORM2-induced increase in proinflammatory factors in monocytes/macrophages and RA-FLSs (Figs. 2, 3), we next questioned whether a cell surface receptor(s) for ORM2 was present in these cells. If so, what receptor could induce cytokine and chemokine production upon ORM2 ligation? To the best of our knowledge, the specific cellular receptor of ORM2 has not been identified. To address this issue, we first utilized previously published protein-to-protein interactome databases (Supplementary Table 2). With these databases, 11 proteins were found to potentially interact with ORM2. Among these 11 proteins, GYPC has been reported to exhibit receptor activity in erythrocytes^29^. Therefore, we next explored whether RA-FLSs and macrophages expressed GYPC. As shown in Supplementary Fig. 6a, GYPC was expressed on peripheral monocytes freshly isolated from healthy donors, as determined by flow cytometry. Notably, the expression of this gene was significantly upregulated by stimulation with IL-1β, TNF-α, or LPS; in fact, a large portion of cultured monocytes (more than 80%) expressed GYPC on their surface (Supplementary Fig. 6a, b). In cultured macrophages differentiated from peripheral monocytes, TNF-α and LPS also significantly upregulated GYPC expression on the surface (Fig. 4a). However, IL-6 stimulation failed to upregulate GYPC expression in macrophages (Fig. 4a).

GYPC was also expressed in RA-FLSs, but its level was much lower than that in cultured monocytes (Fig. 4b). Like in peripheral monocytes, IL-1β and TNF-α, but not LPS or IL-6, upregulated GYPC expression in RA-FLSs (Fig. 4b and Supplementary Fig. 6c). RA-FLSs exhibit heterogeneity and consist of more than three different subtypes, including CD90^+^ and CD55^+^ FLSs^30^. In particular, CD55^+^ FLSs, located in the synovial lining, are involved in bone and cartilage damage and have a limited impact on inflammation, while CD90^+^ fibroblasts in the sublining layer induce more severe inflammation with minimal effects on bone and cartilage^30^. Immunofluorescence staining of RA synovial tissues revealed that GYPC-expressing cells colocalized well with CD68^+^ and CD90^+^ cells, indicating that synovial macrophages and sublining CD90^+^ FLSs are the major cell types that express GYPC (Fig. 4c and Supplementary Fig. 7). In contrast, only a modest percentage of the CD55^+^ cells in the lining layer expressed GYPC (Supplementary Fig. 7), suggesting that these cells are not the major FLS subtype that responds to GYPC stimulation. Furthermore, the colocalization of GYPC with macrophage or FLS markers varied (Supplementary Fig. 7), which might be due to the difference in GYPC expression levels depending on proinflammatory stimuli (Fig. 4a, b).

To test whether GYPC could actually mediate ORM2-induced IL-6 and CXCL8 expression, we further carried out knockdown experiments using *GYPC* siRNA. As a result, *GYPC* siRNA, but not the control siRNA, almost completely blocked the ORM2-induced increase in *IL6* and *CXCL8* mRNA expression in RA-FLSs (Fig. 4d, e). Secretion of IL-6 and CXCL8 from RA-FLSs in the presence of recombinant human ORM2 was also markedly hampered by *GYPC* siRNA (Fig. 4f). In contrast, TNF-α-induced *IL6*, and *CXCL8* expression was rarely affected by *GYPC* siRNA, which excluded the possibility of nonspecific cellular toxicity caused by *GYPC* siRNA (Fig. 4g). Collectively, these results strongly suggest that GYPC can function as an ORM2 receptor in RA-FLSs.

**Fig. 5:**
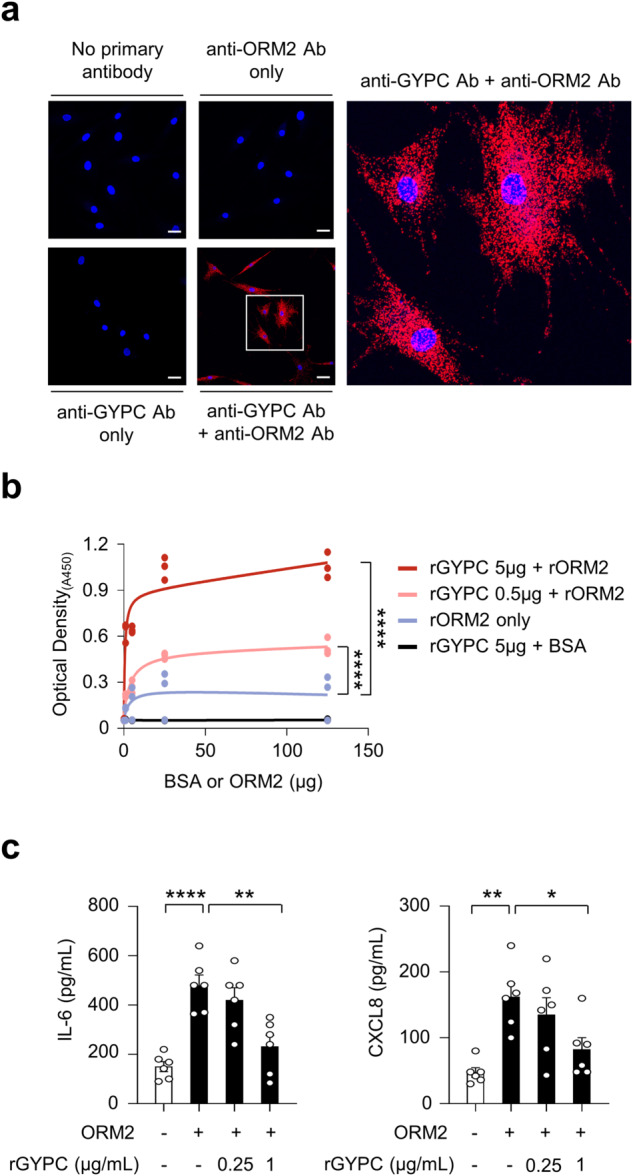
Interaction of ORM2 with its receptor GYPC. **a** Proximity ligation assays of RA-FLSs treated with recombinant ORM2 (1 μg/mL). The red fluorescent dots indicate sites at which the ORM2 and GYPC proteins interact on RA-FLSs. The rectangular area in the middle panel (scale bars: 50 μm) is magnified to the right panel (scale bar: 10 μm). **b** ELISA showing the specific binding of ORM2 to GYPC. The binding plates were coated with recombinant GYPC (rGYPC: 0, 0.5, or 5 μg) and then treated with different amounts (0, 5, 25, or 125 μg) of recombinant ORM2 (rORM2) or bovine serum albumin (BSA). The ‘rORM2 only’ indicates the ELISA results performed with rORM2 at 0, 5, 25, and 125 μg in the absence of the rGYPC coating. **c** Inhibition of IL-6 and CXCL8 production by the soluble form of GYPC. The cells were pretreated with (soluble) recombinant GYPC (rGYPC: 0.25 and 1 μg/mL) for 1 h and then stimulated with 1 μg/mL recombinant ORM2 (rORM2) for 48 h. IL-6 and CXCL8 levels in the culture supernatants were measured via ELISA. *P < 0.05, **P < 0.01, and ****P < 0.0001. The bar graph in (**c**) indicates the mean ± SEM of more than three independent experiments; the *P* values were determined by two-way ANOVA (*P* < 0.0001) for (**b**) and one-way ANOVA (IL-6: *P* < 0.0001; CXCL8: *P* = 0.0029) with Tukey’s multiple comparisons test for (**c**).

To test this hypothesis, we first conducted a proximity ligation assay (PLA) using anti-ORM2 and anti-GYPC antibodies. PLA is a powerful experimental tool that facilitates the detection of protein interactions in situ with high specificity and sensitivity^31^. Robust red fluorescence was observed only when these two antibodies were simultaneously used to treat RA-FLSs in the presence of recombinant ORM2. Indeed, almost all of the observed RA-FLSs exhibited strong fluorescent signals (*see* Materials and Methods for details) (Fig. 5a). Such robust red fluorescence was not observed after a single treatment with either the anti-ORM2 or the anti-GYPC Ab. Moreover, in the absence of recombinant ORM2, although fluorescent signals were noted, their intensity was relatively modest (Supplementary Fig. 8, bottom panel). These findings demonstrated that there may be direct molecular interactions between exogenous ORM2 and GYPC on RA-FLSs. To confirm this, solid-phase ELISA was performed using recombinant GYPC (rGYPC) and recombinant ORM2 (rORM2), in which rGYPC was used to precoat the ELISA plate and rORM2 or bovine serum albumin (BSA) was subsequently added to the plate to react with GYPC. As a result, we observed a dose-dependent increase in optical density with increasing concentrations of rORM2, which was not observed with BSA (Fig. 5b). The optical density was further enhanced by increasing the amount of rGYPC coating on the plate (Fig. 5b), indicating that there was a direct molecular interaction between rGYPC and rORM2. We finally investigated whether the increase in cytokine and chemokine production induced by ORM2 could be blocked by the soluble form of rGYPC. As shown in Fig. 5c, the ORM2-induced increase in IL-6 and CXCL8 secretion by RA-FLSs was substantially mitigated by rGYPC pretreatment. This reduction was dose dependent. Taken together, these results, along with the flow cytometric analysis of GYPC and the PLA data, suggest that the soluble form of rGYPC interferes with the interaction between exogenous ORM2 and the membrane form of GYPC, which leads to the inhibition of IL-6 and CXCL8 upregulation induced by ORM2.

In summary, GYPC, a membrane glycoprotein required for erythrocyte shaping and stability^29^, is expressed in synovial macrophages and RA-FLSs. It can interact with ORM2 as a cell surface receptor to mediate the production of proinflammatory cytokines and chemokines.

### ORM2 induces proinflammatory cytokine production by mouse macrophages and FLSs

**Fig. 6:**
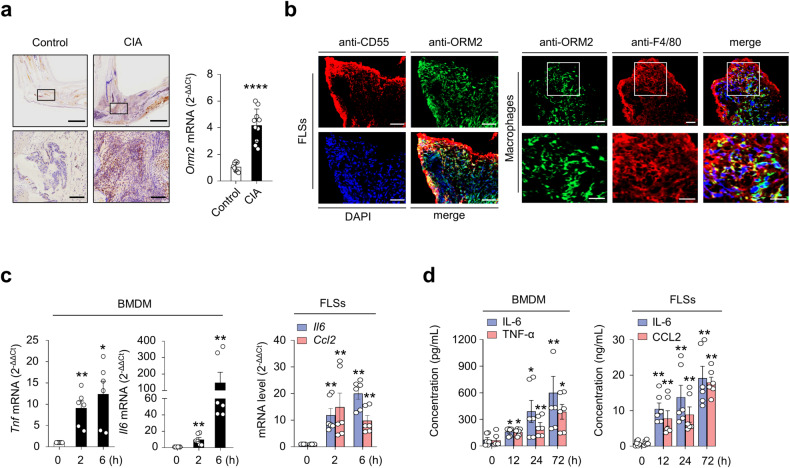
ORM2 expression in mouse synovial tissues and its role in cytokine production. **a** Upregulated ORM2 expression in the synovial tissues of mice with collagen-induced arthritis (CIA). Mice treated with vehicle alone were used as controls. Left panel: Immunohistochemical staining of arthritic joints from mice with CIA using an anti-ORM2 Ab. The rectangular area in the top panel (scale bars: 1000 μm) is magnified to the bottom panel (scale bars: 200 μm). Right panel: qRT‒PCR analysis of *Orm2* expression in the synovia of mice with CIA (*n* = 10) and control mice (*n* = 8). The data are presented as the mean ± SD. ****P < 0.0001 versus control mice according to Welch’s *t* test. **b** Double immunofluorescence staining of synovial tissues from mice with CIA using an anti-ORM2 Ab, an anti-CD55 Ab (for synovial fibroblasts), and an anti-F4/80 Ab (for synovial macrophages). In the merged images, ORM2^+^ cells costained with an anti-CD55 Ab or an anti-F4/80 Ab are shown in yellow. The rectangular area in the upper panel is magnified to the lower panel. Scale bars: 50 μm. For additional immunofluorescence staining data, see Supplementary Fig. 9, which includes synovium staining for two other mice with CIA. **c** ORM2 upregulated *Tnf*, *Il6*, and *Ccl2* expression in mouse BMDMs and FLSs. The cells were stimulated with mouse ORM2 (1 μg/mL) for 2 or 6 h and then subjected to qRT‒PCR. *P < 0.05 and **P < 0.01 versus untreated cells. **d** IL-6, TNF-α, and CCL2 secretion by ORM2-stimulated mouse BMDMs and FLSs was determined via ELISA. The cells were stimulated with mouse ORM2 for 12, 24, or 72 h. *P < 0.05 and **P < 0.01 versus untreated cells. The data in (**c**) and (**d**) are presented as the mean ± SEM of more than three independent experiments; the *P* values were calculated by Brown-Forsythe and Welch ANOVA (*P* = 0.0023) with Dunnett T3 multiple-comparison test for TNF-α secretion by BMDMs in (**c**) and Kruskal–Wallis test (*Il6* by BMDMs in (**c**): *P* < 0.0001; *Il6* by FLSs in (**c**): *P* < 0.0001; *Ccl2* by FLSs in (**c**): *P* = 0.0005; IL-6 by BMDMs in (**d**): *P* = 0.003; TNF-α by BMDMs in (**d**): *P* = 0.0081; IL-6 by FLSs in (**d**): *P* = 0.0014; and CCL2 by FLSs in (**d**): *P* = 0.0004) with post hoc pairwise comparisons test using a Mann–Whitney U test.

Based on human data, we examined whether the regulation of cytokine/chemokine expression by ORM2 could be reproduced in a mouse model. As shown in Fig. 6a, the mean ORM2 expression was much higher (4.2-fold) in mice with collagen-induced arthritis (CIA), a representative animal model of RA^32^, than in control (vehicle-treated) mice, as determined by immunohistochemistry and qPCR analysis of synovial tissues (Fig. 6a). Immunofluorescence staining of the affected joints revealed that, similar to what was observed in RA synovium, ORM2 was strongly colocalized with CD55^+^ and F4/80^+^ cells in the affected joints of mice with CIA (Fig. 6b and Supplementary Fig. 9), confirming that synovial fibroblasts and macrophages are the major cells that produce ORM2. To validate the regulatory effect of ORM2 on the proinflammatory response in the mouse system, mouse bone marrow-derived macrophages (BMDMs) and synovial fibroblasts isolated from the affected joints of mice with CIA were treated with recombinant mouse ORM2. We found that recombinant mouse ORM2 dramatically increased *Tnf* and *Il6* mRNA expression levels in BMDMs in a time-dependent manner (Fig. 6c). After treatment with 1 μg/mL mouse ORM2 for 6 h, the fold change relative to that in the medium alone was 12.4 for *Tnf* and 147.9 for *Il6* (Fig. 6c). Similarly, compared with FLSs treated with medium alone, ORM2-stimulated mouse FLSs exhibited marked increases in *Il6* and *Ccl2* expression levels up to 14.5- and 16.0-fold, respectively (Fig. 6c). However, ORM2 stimulation failed to upregulate *Il8* expression in mouse FLSs (data not shown), unlike in RA-FLSs. Moreover, ORM2 stimulation substantially increased the secretion of IL-6 and TNF-α in mouse BMDMs and of IL-6 and CCL2 in RA-FLSs in a time-dependent manner (Fig. 6d).

Taken together, our results demonstrate that ORM2 is also highly expressed in the inflamed joints of mice, particularly in synovial fibroblasts and macrophages, and directly promotes the secretion of proinflammatory cytokines and chemokines by these cells.

### ORM2 promotes IL-1β-induced arthritis in vivo and reflects the inflammatory activity of RA

**Fig. 7:**
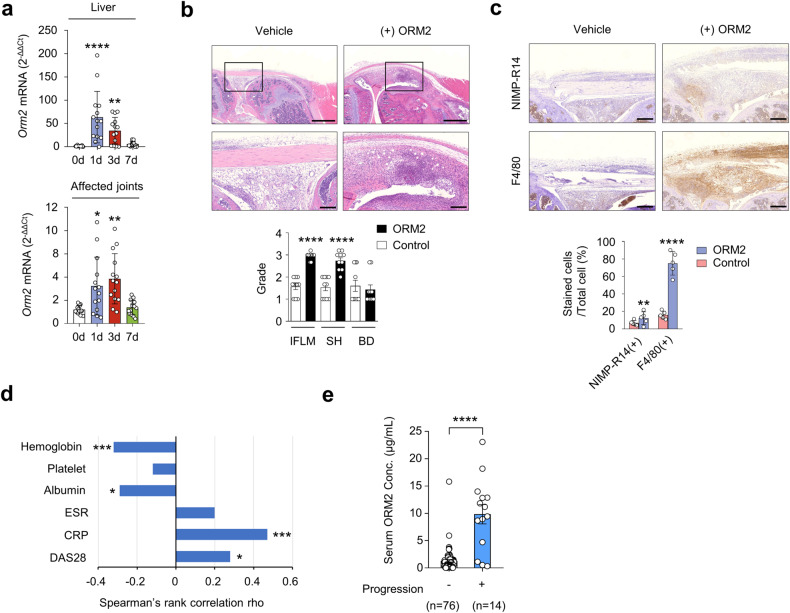
In vivo effect and clinical significance of ORM2 in chronic arthritis. **a** Dynamic expression of *Orm2* in the liver and joints of mice with IL-1β-induced arthritis. After inducing arthritis with IL-1β, the liver (top) and affected joints (bottom) of the mice were harvested on days (d) 0, 1, 3, and 7 and then subjected to qRT‒PCR. The bar graphs represent the mean ± SD. *P < 0.05, **P < 0.01, and ****P < 0.0001 versus Day 0 without arthritis according to the Kruskal–Wallis test (*P* < 0.0001) with Dunn’s multiple comparisons test for the liver and Brown-Forsythe and Welch ANOVA (*P* = 0.0003) with the Dunnett T3 multiple-comparison test for the affected joint. **b** Increased arthritis severity in mice with ORM2-accelerated arthritis (*n* = 10) compared to mice with IL-1β-induced arthritis only (control mice), as determined by the histological grade of the affected knee joint on Day 7. ORM2-accelerated arthritis was generated by injecting recombinant mouse ORM2 (4 μg) into the ipsilateral knee joint of mice with IL-1β-induced arthritis. IFLM, inflammation; SH, synovial hyperplasia; BD, bone destruction. The rectangular area in the upper panel (scale bars: 1000 μm) is magnified to the bottom panel (scale bars: 200 μm). **c** Immunohistochemical staining of the affected knee joints of mice with ORM2-accelerated arthritis versus control mice using an anti-NIMP-R14 Ab and an anti-F4/80 Ab. The number of cells positive for each antibody was manually counted. Scale bars: 200 μm. The data are presented as the mean ± SD. ***P < 0.001 and ****P < 0.0001 versus control mice according to the Mann‒Whitney U test for (**b**) and an unpaired two-tailed t test for (**c**). **d** Spearman’s rank correlations of the serum ORM2 concentration with the blood inflammatory marker levels in RA patients. ESR, erythrocyte sedimentation rate; CRP, C-reactive protein; DAS28, Disease Activity Scale with 28-joint assessment. (**e**) Serum ORM2 concentration (Conc.) according to the radiographic severity of RA. Disease severity was assessed by evaluating radiographic damage via X-rays of the hands and feet, which were taken at baseline and annually thereafter. The statistical analysis in (**d**) and (**e**) was performed with Spearman’s correlation coefficient test: *P < 0.05, ***P < 0.001, and ****P < 0.0001.

Since ORM2 has strong proinflammatory activity in vitro, we explored whether ORM2 could aggravate the severity of chronic arthritis in vivo. To this end, we generated a severe form of ORM2-accelerated arthritis by intra-articular administration of ORM2 (4 μg) into the knee joints of mice with suboptimal IL-1β-induced arthritis (Supplementary Fig. 10a), a model of chronic arthritis in which macrophages play a central role^33^. After inducing IL-1β-induced arthritis in mice, the *Orm2* mRNA expression level increased up to 65.3-fold and 3.2-fold in the liver and affected joints, respectively, as determined by qPCR (Fig. 7a). Moreover, compared with vehicle injection, intra-articular injection of ORM2 markedly exacerbated the suboptimal severity of IL-1β-induced arthritis, as assessed by inflammatory cell infiltration and synovial hyperplasia (Fig. 7b). Interestingly, the infiltrated cells were strongly stained with the anti-F4/80 Ab but only modestly stained with the anti-NIMP-R14 Ab (Fig. 7c and Supplementary Fig. 10b). These findings suggest that the major cell types recruited by ORM2 injection are monocytes/macrophages, in accordance with the in vitro findings that ORM2 increases the CCL2 production necessary for monocyte recruitment^34^, in contrast with the finding that ORM2 has no effect on CXCL8 production for neutrophil recruitment^35^.

Finally, we investigated whether the serum ORM2 concentration could represent inflammatory activity and disease severity in RA patients (*n* = 90) (Supplementary Table 1). The results showed that the serum ORM2 concentration was positively correlated with simultaneously measured parameters for RA activity, including the CRP level (rho = 0.47) and disease activity score 28 (DAS28, rho = 0.28), while it was negatively correlated with the serum ALB concentration (rho = −0.29) and Hb concentration (rho = −0.32) (Fig. 7d). Moreover, serum ORM2 concentrations at baseline were significantly higher in the subgroup of RA patients (*n* = 14) with radiographic progression than in those without such progression (*n* = 76), which was determined using the serial X-ray images from patients collected over 2 years (median serum ORM2 levels [IQR]: 10,310 [4,710-12,870] ng/mL for the progression group and 1,090 [340-1,545] ng/mL for the nonprogression group; *P* < 0.001) (Fig. 7e). Taken together, these results suggest that the serum ORM2 concentration could indicate disease activity and progression in RA patients, suggesting that ORM2 has potential use as a diagnostic marker for RA.

## Discussion

The involvement of acute phase proteins in chronic inflammatory diseases has not been systematically analyzed. Our preliminary data on the global proteome profiles of serum and urine reveal that ORM2, one of the acute phase reactants differentially expressed in RA, showing the highest fold change. Interestingly, ORM2 expression was upregulated in the sera and SFs of RA patients than in those of OA patients. The major cell types producing ORM2 in the joints were synovial macrophages and FLSs. Proinflammatory stimuli substantially increased the secretion of ORM2 by RA synovial macrophages and RA-FLSs. Notably, recombinant ORM2 directly promoted the production of proinflammatory cytokines, including IL-6, CXCL8, CCL2, and TNF-α, by macrophages and RA-FLSs. These increases were mediated through the NF-κB and p38 MAP kinase pathways after ORM2 interacted with its receptor GYPC. In the arthritic joints of mice with CIA, ORM2 expression was upregulated, particularly in synovial macrophages and fibroblasts. ORM2 also directly increased the production of TNF-α, IL-6, and CCL2 in mouse BMDMs and FLSs. Moreover, it increased the pathological severity of IL-1β-induced arthritis in mice and accelerated the infiltration of macrophages in the affected joints. In RA patients, circulating ORM2 levels correlated with disease activity and radiographic progression. Taken together, these results indicate that the acute phase protein ORM2 can directly promote rheumatoid inflammation. Thus, it could be a diagnostic and therapeutic target.

ORM2 has been implicated in autoimmune and infectious diseases, including adult-onset Still’s disease, hepatitis B virus-induced acute liver failure, and tuberculosis^36–38^. In the present study, we conducted an integrative analysis of the serum and urine proteomes of RA patients and suggested that ORM2 is involved in rheumatoid inflammation as an acute phase protein. ORM2 is mainly synthesized in the liver and is subsequently secreted into the blood under infectious and inflammatory conditions^3^. However, the level of ORM2 produced at extrahepatic sites, including macrophages and synoviocytes, is largely unknown. Here, for the first time, we showed that macrophages and FLSs can generate large amounts of ORM2, particularly when they are activated by proinflammatory cytokines and LPS, a Toll-like receptor 4 ligand, clearly demonstrating that these cells are major extrahepatic sources of ORM2 production and explaining the elevated ORM2 expression levels in RA joints. Strikingly, recombinant ORM2 markedly induced proinflammatory responses in macrophages and FLSs in both human and mouse systems, suggesting that secreted ORM2 could in turn activate neighboring macrophages and FLSs in a paracrine and autocrine manner.

GYPC is a membrane glycoprotein required for erythrocyte shaping and mechanical stability^29^. ORM2 was selected as a potential receptor for RA-FLSs on the basis of the protein‒protein interaction database. GYPC is a receptor for EBL-1, a *Plasmodium falciparum* protein in erythrocytes^39^. Moreover, it appears to be broadly expressed in both erythroid and nonerythroid tissues^40^. However, its expression has not yet been demonstrated in joints. Our data showed that GYPC expression in synovial macrophages and in CD90^+^ FLSs located in the sublining of the synovium in RA patients was evident and could be upregulated by inflammatory stimuli, including IL-1β, TNF-α, and/or LPS. Moreover, flow cytometry, proximity ligation assays, *GYPC* knockdown experiments, ELISAs using rGYPC and rORM2, and blocking experiments with soluble rGYPC all support the notion that GYPC functions as a receptor for ORM2 on RA-FLSs to mediate IL-6 and CXCL8 production, which suggests a novel proinflammatory function of GYPC. However, how GYPC transmits its activation signal to upregulate IL-6 and CXCL8 expression remains unclear. GYPC consists of a single extracellular, transmembrane, and cytoplasmic domain^41^. An earlier study demonstrated that an anti-GYPC Ab can inhibit the activity of ERK1/2, a protein kinase^42^, suggesting that GYPC can regulate intracellular kinase activity. Given that NF-κB and p38 MAP kinase are responsible for ORM2-mediated regulation of IL-6 and CXCL8 production, we presume that these signaling pathways can link GYPC receptor activation to cytokine expression in ORM2-stimulated RA-FLSs. However, further studies are needed to clarify this issue.

A variety of liver-originating acute phase proteins, including ORM2, are released into the periphery, where they can be proinflammatory or anti-inflammatory. Indeed, the liver produces diverse acute-phase proteins with anti-inflammatory activities, including α-1 antitrypsin (which inhibits neutrophil elastase)^43^, CRPs^44^, haptoglobin (which suppresses oxidative stress)^45^, and hemopexin (which inhibits the Th17 response)^9^. Our in vivo and translational data provided additional evidence that ORM2 plays a direct proinflammatory role in RA. We demonstrated that an intra-articular injection of recombinant ORM2 into affected joints aggravated the severity of IL-1β-induced arthritis in mice, accompanied by a marked increase in macrophage infiltration. Given the in vitro mouse data, we suspect that this aggravation might be caused by ORM2-induced increases in IL-6, TNF-α, and CCL2 production by IL-1β-activated macrophages and/or FLSs. In parallel, we demonstrated that the serum ORM2 concentration was correlated with the CRP concentration and DAS28 score, suggesting that the ORM2 could reflect the disease activity of RA.

The present study proposed that ORM2-mediated reciprocal activation between macrophages and FLSs accelerates rheumatoid inflammation (Supplementary Fig. 11). As shown in the present study, ORM2 is produced mainly by macrophages and FLSs, particularly after stimulation with TNF-α, IL-1, or a Toll-like receptor-4 agonist. Secreted ORM2 in turn may stimulate the production of proinflammatory cytokines (e.g., IL-6 and TNF-α) and chemokines (e.g., CCL2 and CXCL8) by macrophages and FLSs (Supplementary Fig. 11). Released cytokines/chemokines can in turn upregulate ORM2 and further promote chronic inflammation by activating neighboring macrophages and FLSs and facilitating monocyte recruitment to inflamed joints, which results in a positive feedback loop of rheumatoid inflammation (Supplementary Fig. 11). Considering that ORM2 was synthesized at markedly greater levels in the liver than in the affected joints of mice with IL-1β-induced arthritis (Fig. 7a), a local form of chronic arthritis, we suspect that liver-derived ORM2 could also participate in this process. For example, during acute inflammation, hepatic ORM2 might contribute to the initiation and/or exacerbation of RA. Moreover, given that serum ORM2 levels change in proportion to RA activity, sustained high RA activity may continuously stimulate the liver to produce ORM2 at high levels, which can enter hypervascular RA joints until it reaches equilibrium, thereby promoting chronic synovitis.

This study has several limitations. First, we observed ORM2-induced exacerbation of IL-1β-induced arthritis over a relatively short period of 7 days. However, it is imperative to validate our primary findings in a long-term chronic arthritis model, such as a model of collagen-induced arthritis. Second, since there is no commercially available neutralizing anti-ORM2 Ab, we could not determine whether targeting ORM2 is of therapeutic benefit and failed to determine how important the ORM2-driven inflammatory network is in RA joint pathology, where many other proinflammatory molecules, including cytokines, are involved. Further studies will be required to clarify these issues.

In summary, our work highlights the importance of the acute phase reactant ORM2 in the reciprocal activation of macrophages and FLSs, ultimately leading to the acceleration of rheumatoid inflammation. The present study also demonstrated the novel proinflammatory activity of GYPC, an ORM2 receptor, in addition to its role in regulating erythrocytes. Upon ORM2 stimulation, NF-κB and p38 MAP kinase are major downstream pathways for the robust production of proinflammatory cytokines and chemokines, including IL-6, TNF-α, CXCL8, and CCL2, by macrophages and RA-FLSs. We also functionally validated the pathological role of ORM2 in vivo and determined the clinical importance of the serum ORM2 concentration for determining RA activity and severity. These findings provide new insight into the pathogenic mechanism of RA and emphasize the importance of ORM2 as a diagnostic marker for RA activity as well as a new target for RA treatment. In this regard, anti-ORM2 blockades (e.g., small molecules or monoclonal Abs) could be novel candidates for the treatment of RA and other inflammatory diseases in which ORM2 plays a key role.

### Supplementary information

Supplemental information

## Data Availability

Reagents and other materials in the context of this manuscript will be shared with investigators from not-for-profit organizations who request them in accordance with institutional guidelines using a simple material transfer agreement.
