# Supplementary material for: The acute phase reactant orosomucoid-2 directly promotes rheumatoid inflammation
Source: Exp Mol Med. 2024 Apr 1;56(4):890–903. doi: 10.1038/s12276-024-01188-0 (PMC11058272; doi:10.1038/s12276-024-01188-0)
Supplement: Supplementary file 1 — Supplemental information [file 12276_2024_1188_MOESM1_ESM.docx]

**Supplementary Information**

**Acute Phase Reactant Orosomucoid-2**

**Directly Promotes Rheumatoid Inflammation**

Ki-Myo Kim^1,2*^, Kang-Gu Lee^1,2*^, Saseong Lee^1*^, Bong-Ki Hong^1^, Heejae Yun^1,2^,

Yune-Jung Park^1,3^, Seung-Ah Yoo^1,2^, and Wan-Uk Kim^1,4^

^1^Center for Integrative Rheumatoid Transcriptomics and Dynamics, The Catholic University of Korea, Seoul, South Korea.

^2^Department of Biomedicine & Health Sciences, College of Medicine, The Catholic University of Korea, Seoul, South Korea

^3^Division of Rheumatology, Department of Internal Medicine, St. Vincent’s Hospital, The Catholic University of Korea, Suwon, South Korea.

^4^Department of Internal Medicine, The Catholic University of Korea, Seoul, South Korea

Corresponding author:

Dr. Wan-Uk Kim, Division of Rheumatology, Department of Internal Medicine, The Catholic University of Korea, School of Medicine, Seoul, 137-701, Korea (Fax: 82-2-2258-7526, E-mail: [wan725@catholic.ac.kr](mailto:wan725@catholic.ac.kr)) or Dr. Seung-Ah Yoo, Department of Biomedicine & Health Sciences, College of Medicine, The Catholic University of Korea, School of Medicine, Seoul, 137-701, Korea (Fax: 82-2-2258-7526, E-mail: [youcap78@hanmail.net](mailto:youcap78@hanmail.net))

*These authors contributed equally to this work.

**Keywords:** Orosomucoid-2, Glycophorin C, Macrophages, Synovial Fibroblasts, Rheumatoid Arthritis

**Running title**: ORM2 promotion of RA

**This file includes:**

**Supplementary Materials & Methods**

**Supplementary References**

**Supplementary Fig. 1.** Heatmap showing the proteins differentially expressed in sera or urine samples of RA patients and also defined as acute phase reactant by Gene Ontology

**Supplementary Fig. 2**. Expression of ORM2 in macrophages and fibroblasts in synovial tissues of RA patients

**Supplementary Fig. 3.** Effect of IL-6, IL-10, and pro-fibrotic cytokine TGFβ and M-CSF on ORM2 expression in macrophages and/or RA-FLSs**.**

**Supplementary Fig. 4.** Effects of ORM2 on cytokine/chemokine production and cell viability in RA-FLSs and macrophages.

**Supplementary Fig. 5.** Involvement of NF-κB and p38 pathways in ORM2-induced cytokine production from macrophages

**Supplementary Fig. 6.** Flow cytometry assessment of GYPC in CD14^+^ monocytes and RA-FLSs.

**Supplementary Fig. 7.** Expression of GYPC in macrophages and fibroblasts in synovial tissues of RA patients

**Supplementary Fig. 8.** Proximity ligation assays for detection of interaction of ORM2 and GYPC

**Supplementary Fig. 9.** ORM2 expression in macrophages and fibroblasts in synovial tissues of mice with collagen-induced arthritis

**Supplementary Fig. 10.** Induction of ORM2-accellerated arthritis in mice

**Supplementary Fig. 11.** Hypothetical model for ORM2-dictated reciprocal activation of macrophages and FLSs in RA

**Supplementary Table 1.** Eleven protein-coding genes interacting with ORM2 protein

**Supplementary Table 2.** Demographic features of patients with rheumatoid arthritis

**Supplementary Methods**

***Isolation and culture of mononuclear cells***

Heparinized peripheral blood samples were aseptically collected from healthy donors after obtaining their informed consent and were diluted 1:1 with RPMI 1640 (Gibco BRL, Grand Island, NY, USA). Synovial fluids of RA patients were collected by arthrocentesis into sterile tubes and then diluted 1:5 with phosphate buffered saline (PBS) immediately after collection. Mononuclear cells in the peripheral blood and synovial fluid were isolated by density gradient centrifugation on Ficoll-Hypaque (Cytiva, Marlborough, MA, USA). Cells were then resuspended in RPMI 1640 medium supplemented with 1% fetal bovine serum (FBS; Gibco BRL), 100 U/mL penicillin, 100 μg/mL streptomycin, and 2 mM L-glutamine. Each culture was performed in triplicate at a density of 1 × 10^6^ cells/well in 96 microtiter wells (SPL Life Sciences, Pocheon-si, Gyeonggi-do, Korea) at 37°C in a 5% CO_2_ atmosphere. In some experiments, CD14^+^ cells were separated from synovial fluid mononuclear cells (SFMC) or peripheral blood mononuclear cells (PBMC) using CD14 microbeads (Miltenyi Biotec, Bergisch Gladbach, Germany). For monocyte-derived macrophages, blood CD14^+^ cells from healthy donors were cultured with M-CSF (216-MC, 20 ng/mL; R&D Systems, Minneapolis, MN, USA) for 3 days. These cells were then cultured in RPMI1640 medium and stimulated with IL-1β (RIL1B1, 10 ng/mL; Thermo Fisher Scientific, Waltham, MA, USA), TNF-α (200-TA , 10 ng/mL; R&D Systems, Minneapolis, MN, USA), lipopolysaccharide (LPS, L2630, 100 ng/mL; Sigma-Aldrich, St. Louis, MO, USA), IL-6 (200-06, 10 ng/mL; Peprotech, Cranbury, NJ, USA), or recombinant ORM2 (PRO-1560; Prospec-Tany TechnoGene Ltd., Rehovot, Israel) for the indicated times. Cell-free supernatants were collected and stored at -20°C until assayed.

***Isolation and culture of synoviocytes***

FLSs were isolated from the synovial tissues of patients with RA and OA as described previously^1^. Briefly, synovial tissues were spliced into 2- to 3-mm pieces and treated with 0.1 mg/mL of type 1 collagenase (Thermo Fisher Scientific) in Dulbecco's Modified Eagle Medium (DMEM) at 37°C in a CO_2_ incubator for 4 hours. Dissociated cells were then resuspended in DMEM supplemented with 10% FBS, 2 mM L-glutamine, penicillin (100 U/mL), and streptomycin (100 μg/mL) and plated in 75-cm^2^ flasks. Cells were then cultured at 37°C with 5% CO_2_. The culture medium was refreshed every three days. When the cells reached confluence, they were passed by diluting 1:3 with fresh medium and re-cultured until used. FLSs from passages 4 through 8 were used for each experiment.

In some experiments, FLSs were stimulated with IL-1β (RIL1B1, 10 ng/mL; Thermo Fisher Scientific), TNF-α (200-TA, 10 ng/mL; R&D Systems), TGF-β (100-21C, 10 ng/mL; R&D systems), lipopolysaccharide (LPS, L2630, 100 ng/mL; Sigma-Aldrich), IL-6 (200-06, 10 ng/mL; Peprotech), M-CSF (216-MC, 10 ng/mL; R&D Systems), IL-10 (217-IL, 10ng/mL; R&D Systems), or recombinant ORM2 (PRO-1560, Prospec-Tany TechnoGene Ltd.). Recombinant ORM2 used was a single polypeptide chain encompassing 208 amino acids (19-201) and had a purity of more than 95% as determined by SDS-PAGE. To eliminate concerns of endotoxin contamination, all culture experiments using recombinant ORM2 were performed in the presence of polymyxin B (30 μg/mL).

***Enzyme-linked immunosorbent assay (ELISA) for ORM2, IL-6, TNF-α, CXCL8, and CCL2***

ORM2 concentrations in the sera, synovial fluids, and culture supernatants were measured using an ELISA kit (Mybiosource, San Diego, CA, USA). Levels of IL-6, TNF-α, CXCL8 (IL-8), CCL2 in the sera, synovial fluids, and culture supernatants were also determined by ELISA (R&D Systems) as described previously^2^.

***Immunohistochemistry for ORM2, NIMP-R14, and F4/80***

Immunohistochemistry staining for ORM2 was performed using RA and OA synovium samples as described previously^1^. Briefly, five μm sections of paraffin-embedded blocks were mounted on superfrost glass slides, deparaffinized in xylene, and rehydrated in a graded series of ethanol, followed by microwave antigen retrieval. The endogenous peroxidase activity was blocked by 2% hydrogen peroxide. After blocking nonspecific binding with 10% normal horse serum at room temperature for 60 minutes, the slides were incubated with rabbit anti-ORM2 Ab (bs-7565R, clone 121-201aa, 1:200; Bioss, Woburn, MA, USA), rat anti-F4/80 Ab for macrophages (MCA497GA, clone Cl:A3-1, 1:200; Bio-Rad, Hercules, CA, USA), or rat anti-NIMP-R14 Ab for neutrophils (sc-59338, 1:100; Santa Cruz Biotechnology, Dallas, TX, USA) at 4°C overnight. These slides were washed with PBS and incubated again with an anti-rabbit or anti-rat secondary Ab using ImmPRESS polymer detection kits (MP-7401, MP-7404, Vector Labs, Burlingame, CA, USA) at room temperature for 1 hour. Isotype control Ab (MAB005, clone AB-105-C, IgG; R&D systems) was used as a control. Slides were then counterstained with Harris hematoxylin, dehydrated, cleared, and mounted.

***Immunofluorescence staining for ORM2 and GYPC***

For immunofluorescence experiments, frozen synovial tissues of RA patients and mice were incubated with anti-CD55 Ab (sc-51733, clone NaM16-4D3, 1:100; Santa Cruz Biotechnology), anti-CD68 Ab (sc-20060, clone KP1, 1:100; Santa Cruz Biotechnology), anti-F4/80 Ab (MCA497GA, clone Cl:A3-1, 1:100; Bio-Rad), anti-ORM2 Ab (bs-7565R, Ab; 1:200; Bioss), anti-CD90 Ab (ab133350, , clone EPR3133, 1:25; Abcam, Cambridge, England), and anti-GYPC (sc-59183, clone BRIC10, 1:50; Santa Cruz Biotechnology). Samples were then stained with Alexa Fluor 488-conjugated anti-IgG (A21202, A21206, Thermo Fisher Scientific) or Alexa Fluor 594-conjugated anti-IgG (A21203, A21207, Thermo Fisher Scientific) at room temperature for 1 hour. Nuclei were stained with DAPI (1:500; BD Biosciences, San Jose, CA, USA). Slides were mounted in a ProLong Antifade solution (Thermo Fisher Scientific). Stained tissues were then visualized with a confocal microscope (ZEISS, LSM800, Gottingen, Germany).

For pre-absorption blocking experiments, anti-ORM2 Ab (bs-7565R, 1 μg, Bioss) was preabsorbed with recombinant human ORM2 (3 μg) in PBS containing 10% normal Donkey serum overnight at 4°C; the mixture of anti-ORM2 Ab and recombinant ORM2 was incubated overnight on a turning wheel. The next day, synovial tissues were incubated with either the resultant pre-absorbed Ab or un-preabsorbed anti-ORM2 Ab (1 μg) overnight at 4°C. The samples were stained with secondary Ab at room temperature for 1 hour. Nuclei were stained with DAPI and the slides were mounted in a ProLong Antifade solution

***Quantitative real-time PCR***

Total RNAs were isolated from cultured cells using an RNeasy Mini Kit (QIAGEN, Hilden, Germany) and then were used to synthesize cDNAs with a RevertAid reverse transcriptase (Thermo Fisher Scientific). Quantitative real-time PCR (qRT-PCR) was performed in a CFX96 real-time PCR system using SYBR Green PCR premix (Bio-Rad). Expression levels of target genes were calculated by the comparative threshold (C_T_) method and normalized by those of GAPDH (internal control). The relative fold of induction was calculated using the 2^-ΔΔCt^ algorithm. The following primers (forward and reverse) were used for PCR amplification: *Human ORM2*, 5’-GCTGTTCCTTAGGGACACCAA-3’ and 5’-TGACATCTGACCTGGGAATGC-3’; *Mouse Orm2*, 5’- TTGTCATGGTGAGCCTCCTG-3’ and 5’- ATGAAGGCCCCA-TGCATCTT-3’; *Human IL6*, 5’- TTTTGCCAAGGAGTGCTA

-AAGA-3’ and 5’- AACCCTC-TGCACCCAGTTTTC-3’; *Mouse Il6*, 5’- TTCCATCCAGTT

-GCCTTCTTG-3’ and 5’- AGGTCTGTTGGGAGTGGTATC-3’; *Human CXCL8*, 5’- TCCAG

-AACAGATTTGAGAGT-3’ and 5’- GCATTTGTGGTTGGGTCAGG-3’; *Human TNF*, 5’-CTTCTCCTTCCTGATCGTGG-3’ and 5’-GCTGGTTATCTCTCAGCTCCA-3’; *Mouse Tnf*, 5’- TGAAGGGAATGGGTGTTCAT-3’ and 5’-TTGGACCCTGAGCCATAATC-3’; *Human CCL2*, 5’- AAGCAGAAGTGGGTTCAGGA-3’ and 5’- GGGGAAAGCTAGGGGAAAAT-3’; *Mouse Ccl2*, 5’- TTCTTCGATTTGGGTCTCCTTG-3’ and 5’-GTGCAGCTCTTGTCGGTG

-AA-3’; *Human GYPC*, 5’- CCATCGTCCTAGTCTCCCTCC-3’ and 5’-CTTGGCCTCATTG

-GTGTGGTA-3’; *Human GAPDH*, 5’-AAGGTGAAGGTCGGAGTCAA-3’ and 5’-AATGAAGGGGTCATTGATGG-3’; and *Mouse Gapdh*, 5’- AGGTCGGTGTGAACGGATT

-TG-3’ and 5’- TGTAGACCATGTAGTTGAGGTCA-3’.

***Western blotting***

FLSs of RA patients (RA-FLSs) were lysed in a lysis buffer, and the insoluble material was removed after centrifugation at 14,000 rpm for 20 minutes at 4°C. Final protein concentrations were determined using a Bradford protein assay (Bio-Rad). Proteins were then subjected to SDS-PAGE and transferred to nitrocellulose membranes. After blocking with 5% skim milk in PBS containing 0.05% Tween 20 (PBST), membranes were incubated with appropriate dilutions of the following antibodies at 4°C overnight: antibodies against ORM2 (bs-7565R, Bioss), NF-κB p65 (ab7970, Abcam), NF-κB phospho-p65 (NF-κB *p*-p65; 3031s, clone Ser536, Cell Signaling, Danvers, MA, USA), p38 MAP kinase (9212s, Cell Signaling), phospho-p38 MAP kinase (*p*-p38 MAP kinase; 9211s, clone Thr183/Tyr182, Cell Signaling), I-κBα (sc-371, clone C-21, Santa Cruz Biotechnology), GYPC (ab108925, Abcam), and β-actin (sc-47778, clone C4, Santa Cruz Biotechnology). Membranes were washed with PBS-Tween 20, incubated with HRP-conjugated anti-mouse IgG (#31430, Thermo Fisher Scientific) or anti-rabbit IgG (170-6515, Bio-Rad) as a secondary Ab, and visualized using an enhanced chemiluminescent detection system (Santa Cruz biotechnology).

***Flow cytometry analysis for GYPC***

To detect GYPC expression on the cell surface, peripheral blood mononuclear cells (PBMCs) and RA-FLSs were stained with mouse anti-human GYPC Ab (sc-59183, clone BRIC10, 1:20; Santa Cruz biotechnology) for 1 hour at 4^o^C. The cells were then incubated with fluorescein isothiocyanate (FITC)-conjugated secondary Ab (A90-116D2, 1:50; Bethyl Laboratories, Montgomery, TX, USA) or phycoerythrin (PE)-conjugated secondary Ab for (12-4010-82, 1:100; Thermo Fisher Scientific) for 30 minutes at 4^o^C. To detect CD14^+^ monocytes in PBMCs, PBMCs were stained again with PE-conjugated anti-human CD14 Ab (12-0149-42, 1:100; Thermo Fisher Scientific) for 30 minutes at 4^o^C. Cells were resuspended in a fluorescence-activated cell sorting (FACS) buffer and analyzed using a FACS Canto II system (BD biosciences) with FlowJo software (BD biosciences). Mouse IgG_1_ Ab (MAB002, R&D system) was used as an isotype control.

**Supplementary references**

1. Kong, J. S. *et al.* Dynamic transcriptome analysis unveils key proresolving factors of chronic inflammatory arthritis. *J Clin Invest* **130**, 3974-3986 (2020).

2. Yoo, S. A. *et al.* Placental growth factor regulates the generation of T(H)17 cells to link angiogenesis with autoimmunity. *Nat Immunol* **20**, 1348-1359 (2019).

**
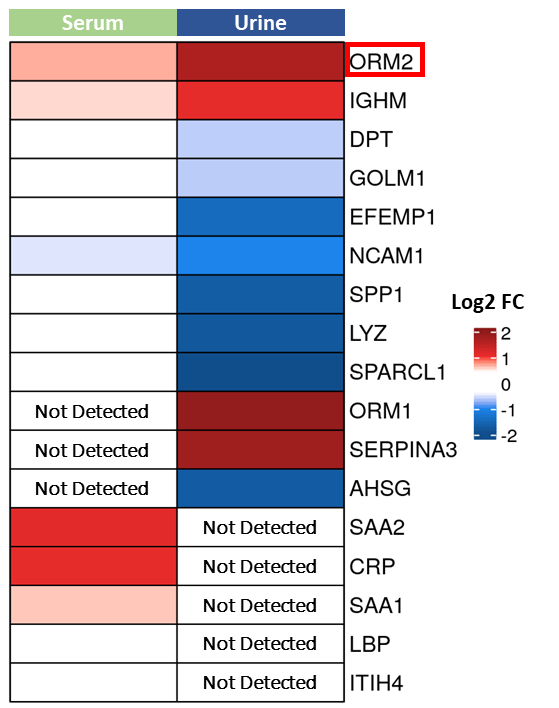
**

**Supplementary Fig. 1. Heatmap showing the proteins differentially expressed in sera or urine samples of RA patients and also defined as acute phase reactant by Gene Ontology.**


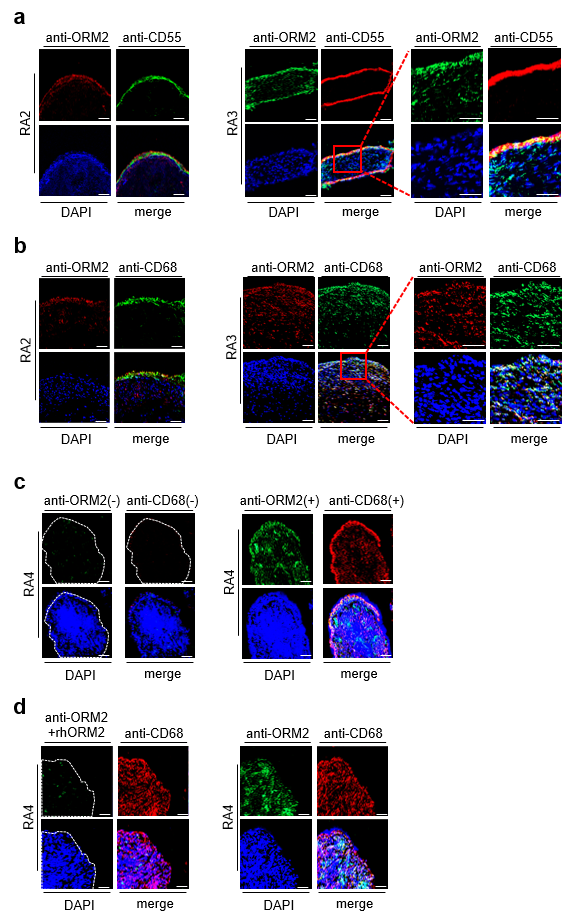


**Supplementary Fig. 2. Expression of ORM2 in macrophages and fibroblasts in synovial tissues of RA patients.** (**a** and **b**) Synovial tissues of two other RA patients (RA 2 and 3), in addition to an RA patient in **Fig. 1d,** were subjected to double immunofluorescence staining using antibodies against ORM2, CD55, and CD68. Scale bars: 50 μm. (**c**) Double immunofluorescence staining of a RA synovial tissue (RA 4) in the absence or presence of anti-ORM2 Ab and anti-CD68 Ab. (**d)** Specificity of anti-ORM2 Ab. Prior to immuno-staining, anti-ORM2 Ab (1 μg) was preabsorbed with recombinant human ORM2 (3 μg) in PBS containing 10% normal donkey serum overnight at 4°C. The RA synovial tissue (RA 4) was then incubated with either the resultant preabsorbed Ab or with un-preabsorbed anti-ORM2 Ab (1 μg) overnight at 4°C. The samples were stained with secondary Ab at room temperature for 1 hour. Nuclei were stained with DAPI.


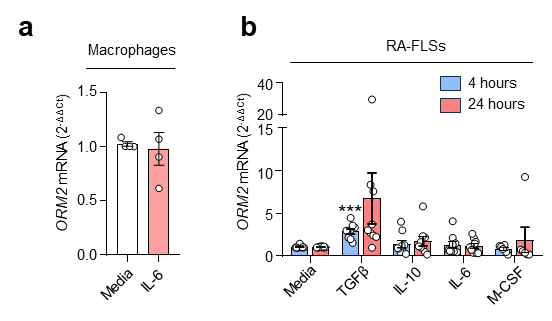


**Supplementary Fig. 3. Effect of IL-6, IL-10, and pro-fibrotic cytokine TGFβ and M-CSF on ORM2 expression in macrophages and/or RA-FLSs.** (**a**) Macrophages (n=4) were differentiated from CD14^+^ monocytes, isolated from peripheral blood of healthy donors, by treating M-CSF (20 ng/mL) for 3 days. The cells were then stimulated with IL-6 (10 ng/mL) for 24 hours. Data are mean ± SEM. Statistical significance was analyzed by Mann-Whitney U test. (**b**) RA-FLSs (n=9) were treated with TGFβ (10 ng/mL), IL-10 (10 ng/mL), IL-6 (10 ng/mL), and M-CSF (10 ng/mL) for the indicated times. ORM2 expression levels were determined by qRT-PCR. Data are mean ± SEM. ****P* < 0.001 by versus media alone by one-way ANOVA (*P* < 0.001) with Dunnett's multiple comparisons test.


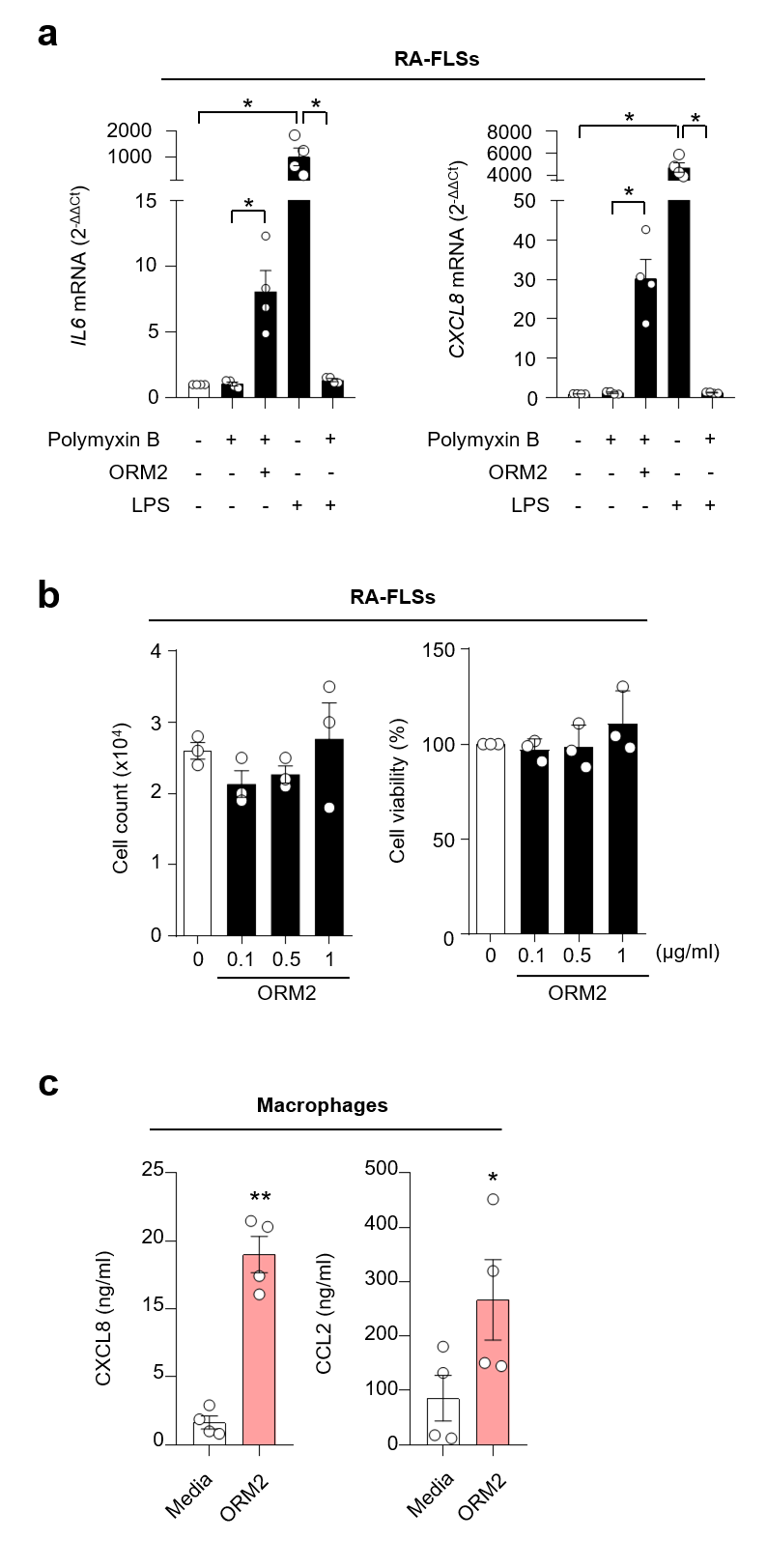
**Supplementary Fig. 4. Effects of ORM2 on cytokine/chemokine production and cell viability in RA-FLSs and macrophages.** (**a**) RA-FLSs (n=4) were cultured in DMEM containing 1% FBS and stimulated with either LPS (100 ng/mL) or recombinant ORM2 (1 μg/mL) in the presence of polymyxin B (30 μg/mL) for 6 hours. *IL6* and *CXCL8* mRNA expression levels were then determined by qRT-PCR. Data are the mean ± SEM. **P* < 0.05 by Kruskal-Wallis test (*IL6*: *P*=0.0027; *CXCL8*: *P*=0.0036) and post hoc pairwise comparison using a Mann–Whitney U test. (**b**) RA-FLSs (n=3) were cultured in DMEM containing 10% FBS and stimulated with recombinant ORM2 at various concentrations (0.1 to 1 μg/mL) in the presence of polymyxin B (30 μg/mL) for 48 hours. Live cell counting and cell viability assessment were conducted through trypan blue exclusion assay (upper panel) and MTT assay (lower panel). Results are presented as means ± SEM. *P* values were analyzed by Brown-Forsythe and Welch ANOVA test (*P*=0.1179) with Dunnett T3 multiple-comparison test for cell count and Kruskal–Wallis test (*P*=0.6375) with Dunn's multiple comparisons test for cell viability. (**c**) Macrophages (n=4) were differentiated from CD14^+^ monocytes, isolated from peripheral blood of healthy donors, by treating M-CSF (20 ng/mL) for 3 days. The cells were then cultured with recombinant ORM2 (1 μg/mL) in the presence of polymyxin B (30 μg/mL) for 24 hours. Levels of CXCL8 and CCL2 were determined by ELISA. Results are presented as means ± SEM. **P* < 0.05, ***P* < 0.01 by paired two-tailed t-test.


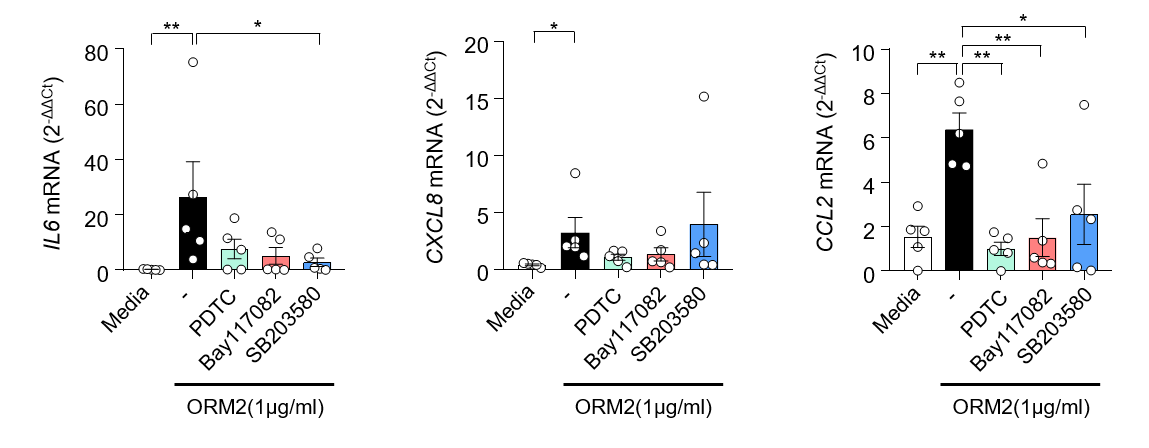


**Supplementary Fig. 5. Involvement of NF-κB and p38 pathways in ORM2-induced cytokine production from macrophages.** Effects of NF-κB and p38 MAP kinase inhibitors on ORM2-stimulated *IL6, CXCL8*, and *CCL2* expression. Macrophages were pretreated with PDTC (10 μM), BAY 117082 (40 μM), and SB203580 (10 μM) for 1 hour and then stimulated with recombinant ORM2 (1 μg/mL) for 6 hours. *IL6, CXCL8,* and *CCL2* mRNA levels were assessed by qRT-PCR. Data represent mean ± SEM of more than three independent experiments. **P* < 0.05 and ***P* < 0.01 Kruskal–Wallis test (*IL6*: *P*=0.0387; *CXCL8*: *P*=0.0605) with post hoc pairwise comparison using a Mann–Whitney U test and one-way ANOVA (*P*=0.0011) with Tukey's multiple comparisons test for CCL2.


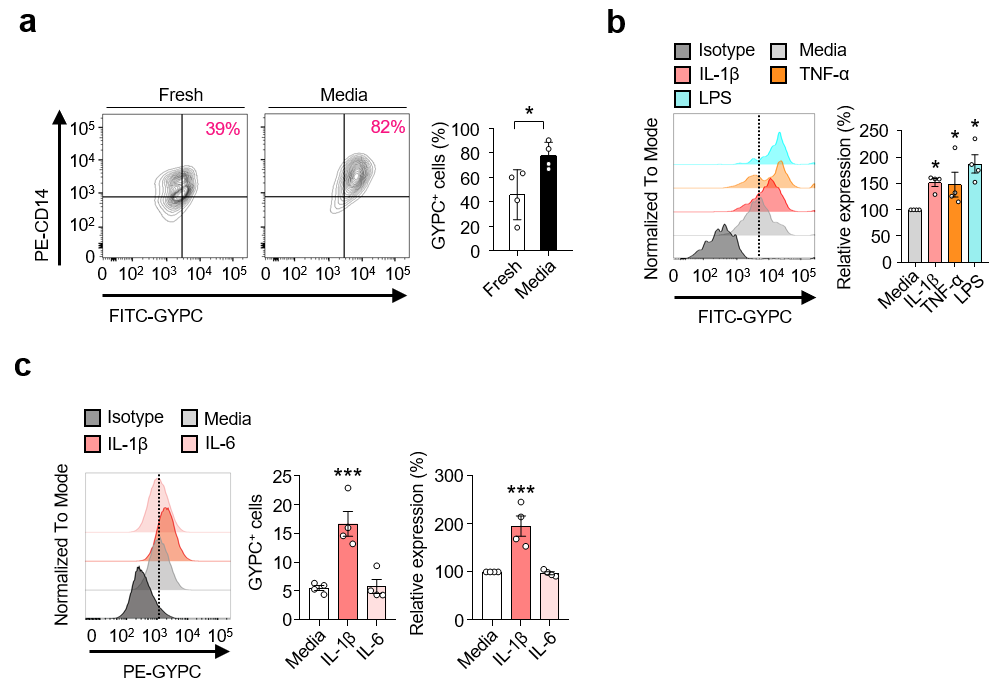


**Supplementary Fig. 6. Flow cytometry assessment of GYPC in CD14^+^ monocytes and RA-FLSs.** (**a** and **b**) Flow cytometry analysis of glycophorin C (GYPC) on cultured monocytes. Peripheral blood mononuclear cells (PBMC) were isolated from heathy donors (n=4) and treated with IL-1β (10 ng/mL), TNF-α (10 ng/mL), and LPS (100 ng/mL) for 24 hours. A representative plot is shown in the left panel. ‘Fresh’ indicates freshly isolated PBMCs and ‘Media’ denotes PBMCs cultured with media alone. Data are mean ± SEM of more than three independent experiments. * *P* < 0.05 versus ‘Fresh’ for (a) and media alone for (b), respectively, by unpaired two-tailed t-test for (a) and Kruskal–Wallis test (*P*=0.0031) with post hoc pairwise comparisons test using a Mann–Whitney U test for (b). (**c**) RA-FLSs (n=4) were treated with IL-1β (10 ng/mL), and IL-6 (10 ng/mL) for 24 hours. A representative plot is shown in the left panel. Data are mean ± SEM. **P* < 0.05, ****P* < 0.0001 by versus media alone by one-way ANOVA (*P* < 0.001) with Dunnett's multiple comparisons test.


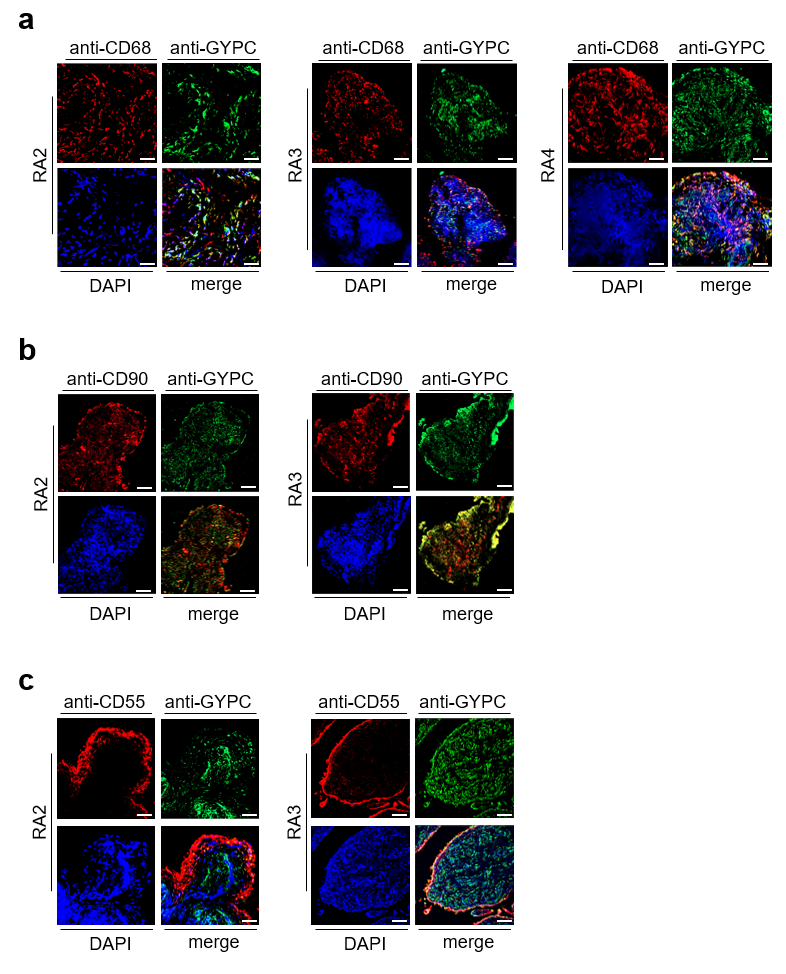


**Supplementary Fig. 7. Expression of GYPC in macrophages and fibroblasts in RA synovial tissues.** Double immunofluorescence staining was performed in synovial tissues of other three RA patients (RA 2, 3, and 4), in addition to an RA patient in **Fig. 4c,** using antibodies against GYPC, CD68, CD90, and CD55. Scale bars in (a), (b), and (c) left panel : 50 μm and in (c) right panel 100 μm.


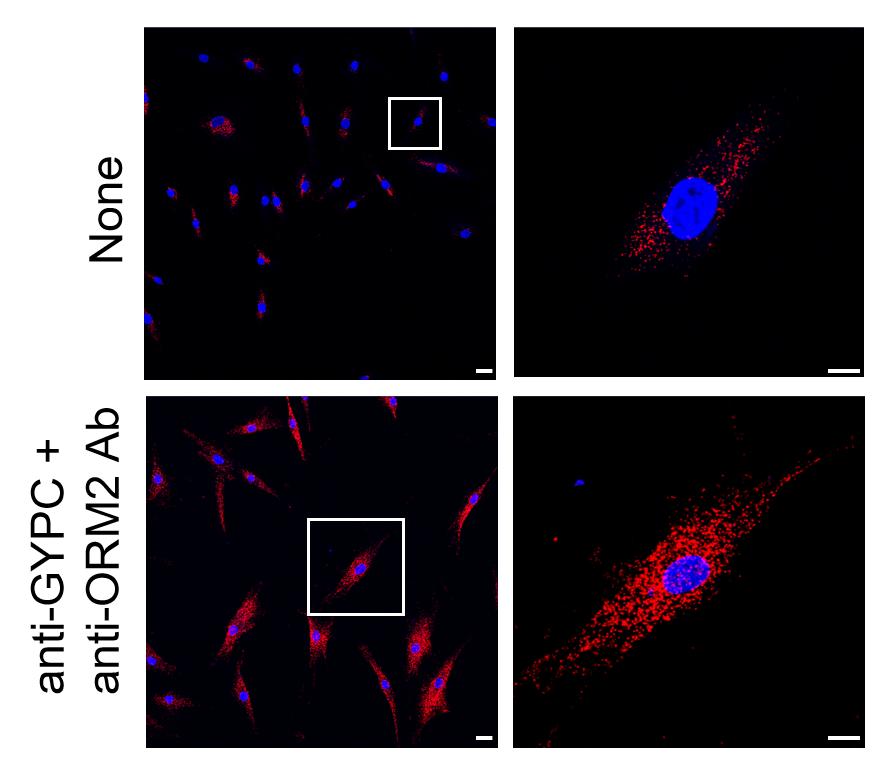


**Supplementary Fig. 8. Proximity ligation assays for detection of interaction of ORM2 and GYPC.** RA-FLSs were treated with anti-ORM2 Ab plus anti-GYPC Ab without recombinant ORM2 (1 μg/mL). Red fluorescent dots represent molecular interaction of endogenous ORM2 produced by RA-FLSs and GYPC. Rectangular area in the left panel (scale bars: 50 μm) is magnified to the right panel (scale bars: 10 μm).


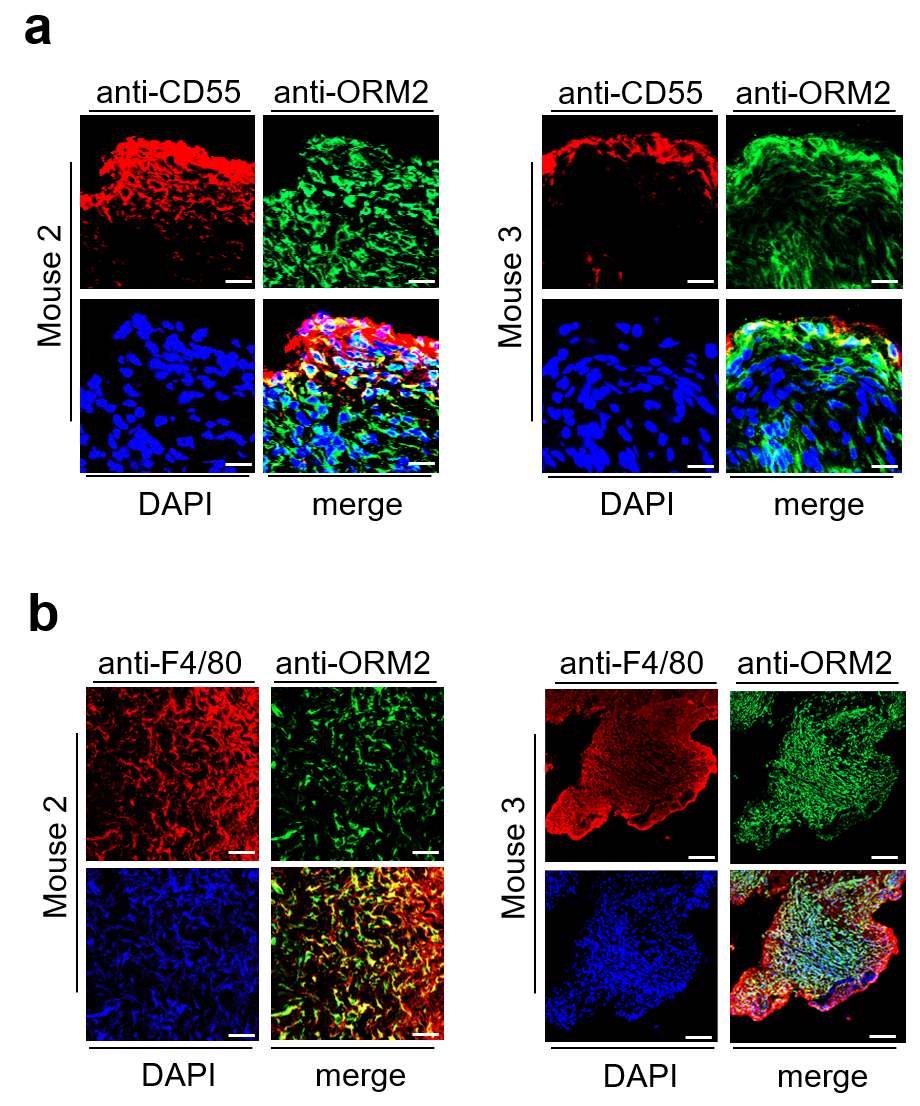


**Supplementary Fig. 9. Expression of ORM2 in macrophages and fibroblasts in synovial tissues of mice with collagen-induced arthritis.** Synovial tissues of two other mice with collagen-induced arthritis (Mouse 2 and 3), in addition to a mouse in **Fig. 6b,** were subjected to double immunofluorescence staining using antibodies against ORM2, CD55 for (a), and F4/80 for (b). Scale bars: 50, 100 or 200 μm.


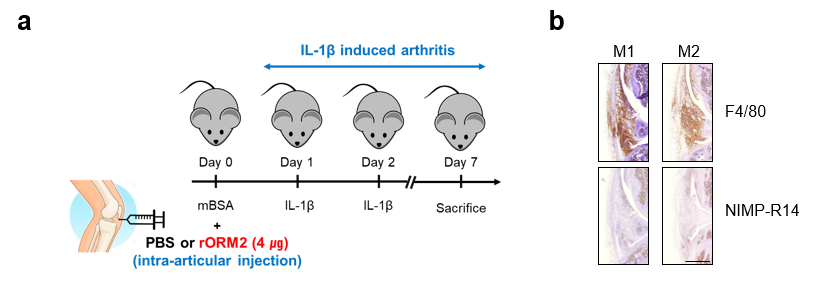


**Supplementary Fig. 10. Induction of ORM2-accellerated arthritis in mice.** (**a**) Experimental scheme of ORM2-accellerated arthritis. Eight-week-old C57BL/6N mice were intra-articularly injected with 10 μL of methylated bovine serum albumin (mBSA) at a concentration of 20 mg/mL into both knee joints on day 0. At the same time, 10 μL of mouse ORM2 at 400 μg/mL was injected into unilateral knee joint on day 0. The opposite knee joint was injected with vehicle alone. Recombinant IL-1β (250 ng) was then subcutaneously injected into the footpad ipsilateral to ORM2 injection site twice on days 1 and 2. Mice were sacrificed on day 7 and subjected to histologic analysis. (**b**) Immunohistochemical staining of affected joints of mice with ORM2-accelerated arthritis was conducted using anti-F4/80 Ab and anti-NIMP-R14 Ab. M1: mouse 1, and M2, mouse 2. Scale bar, 500 μm.


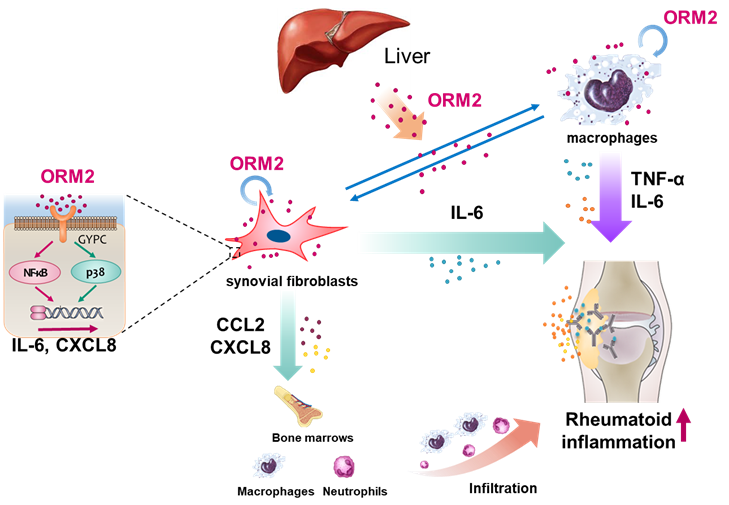


**Supplementary Fig. 11. Hypothetical model for ORM2-dictated reciprocal activation of macrophages and FLSs in RA.** Under high inflammatory conditions, ORM2 is actively produced from arthritic joints, particularly by stimulation of synovial macrophages and fibroblasts with TNF-α, IL-1, and Toll-like receptor-4 agonists, as well as from the liver. The secreted ORM2 in turn binds to a functional receptor GYPC on RA-FLSs, activates NF-κB and p38 MAP kinase pathways, and then directly induces production of pro-inflammatory cytokines (e.g., IL-6, TNF-α) and chemokines (e.g., CXCL8, CCL2) via the two signaling pathways. Resultant cytokines and chemokine can amplify chronic inflammation by further activating macrophages and FLSs and by facilitating recruitment of monocytes and neutrophils to inflamed joints. As a result, more activated macrophages and FLSs can secrete greater amounts of ORM2 and thereby lead to ORM2-centered mutual activation of macrophages and FLSs, constructing a feed-forward cycle of rheumatoid inflammation.

**Supplementary Table 1. Demographic features of patients with rheumatoid arthritis**

|  | Non-progression | Progression | *P*-value^*^ |
| --- | --- | --- | --- |
|  | (n=76) | (n=14) |  |
| Age, year | 60.0 [49.5;66.5] | 55.0 [52.0;60.0] | 0.179 |
| Female, n | 62 (81.6%) | 13 (92.9%) | 0.450 |
| Body mass index, kg/m^2^ | 23.0 [21.0;25.2] | 22.4 [19.8;23.4] | 0.180 |
| Diabetes mellitus, n | 4 (5.3%) | 1 (7.1%) | 0.584 |
| Hypertension, n | 12 (16.0%) | 3 (21.4%) | 0.698 |
| Smoking, n | 3 (4.0%) | 1 (7.1%) | 0.502 |
| Hemoglobin, mg/dl | 12.8 ±1.5 | 12.2 ±1.5 | 0.202 |
| Disease duration, year | 5.5 [1.5;12.5] | 13.0 [3.0;18.0] | 0.215 |
| Albumin, mg/dl | 4.0 [3.8; 4.2] | 4.0 [3.5; 4.4] | 0.973 |
| Cr, mg/dl | 0.7 [0.6; 0.8] | 0.7 [0.6; 0.7] | 0.148 |
| Total Cholesterol, mg/dl | 189.4 ± 36.8 | 179.9 ± 32.1 | 0.368 |
| Triglyceride, mg/dl | 92.0 [65.5;143.0] | 86.5 [66.0;120.0] | 0.589 |
| ESR, mm/hour | 34.5 [18.5;45.0] | 34.5 [24.0;53.0] | 0.577 |
| CRP, mg/dl | 0.4 [ 0.1; 0.9] | 0.4 [ 0.1; 1.4] | 0.730 |
| RF, n^†^ | 65 (85.5%) | 11 (78.6%) | 0.451 |
| Anti-CCP, n^†^ | 62 (91.2%) | 10 (76.9%) | 0.153 |
| DAS28 | 3.6 [ 3.3; 4.8] | 3.4 [ 3.1; 5.4] | 0.830 |
| TJC, n | 1.0 [ 0.0; 4.0] | 1.0 [ 0.0; 4.0] | 0.921 |
| SJC, n | 1.0 [ 0.0; 2.0] | 0.0 [ 0.0; 5.0] | 0.851 |
| Prednisolone, n | 62 (81.6%) | 13 (92.9%) | 0.450 |
| Methotrexate, n | 54 (71.1%) | 13 (92.9%) | 0.105 |
| Hydroxychloroquine, n | 37 (48.7%) | 9 (64.3%) | 0.434 |
| Sulfasalazine, n | 10 (13.2%) | 5 (35.7%) | 0.053 |
| Leflunomide, n | 34 (44.7%) | 7 (50.0%) | 0.943 |
| Anti-TNF-ɑ, n | 6 (7.9%) | 3 (21.4%) | 0.121 |
| Baseline SvdH score | 16.5 [ 8.0;39.0] | 18.5 [ 5.0;57.0] | 0.907 |
| Erosion | 8.0 [ 1.0;15.0] | 5.0 [ 2.0;17.0] | 0.929 |
| JSN | 9.0 [ 5.5;22.0] | 13.0 [ 3.0;30.0] | 0.656 |

Data are presented as the mean ± SD, median [interquartile range], or number (percentage) as appropriate. ESR, Erythrocyte sedimentation rate; CRP, C-reactive protein; RF, rheumatoid factor (positive at 15 IU/mL); Anti-CCP, anti-cyclic citrullinated peptide antibody (positive at 5> units/mL); DAS28, Disease activity score in 28 joints; TJC, tender joint count; SJC, swollen joint count; TNF-ɑ, tumor necrosis factor-ɑ; SvdH, modified Sharp van der Heijde; JSN, joint space narrowing.

* Differences between mean values were examined by Student’s t-test. Nonparametric data were compared between groups using the Mann-Whitney U test. For categorical data, chi-square test was performed. If warning present, Fisher test was performed. † Antibody positivity.

**Supplementary Table 2. Eleven protein-coding genes interacting with ORM2 protein (**protein-coding genes localized in plasma membrane or extracellular region are shown)

| ***Entrez ID** | **Symbol** | **Description** | **Plasma membrane**  **/Extracellular region** | **Receptor Activity** |
| --- | --- | --- | --- | --- |
| 1095 | CEACAMP7 | carcinoembryonic antigen-related cell adhesion molecule pseudogene 7 | - |  |
| 2995 | GYPC | glycophorin C  (Gerbich blood group) | O | Lobo CA et al. 2003  (ref. 16) |
| 4204 | MECP2 | methyl-CpG binding protein 2 | O |  |
| 6788 | STK3 | serine/threonine kinase 3 | - |  |
| 7168 | CMH3 | cardiomyopathy, hypertrophic 3 | - |  |
| 7521 | XRCC8 | X-ray repair complementing defective repair in Chinese hamster cells 8 | - |  |
| 8916 | HERC3 | HECT and RLD domain containing E3 ubiquitin protein ligase 3 | - |  |
| 9588 | PRDX6 | peroxiredoxin 6 | O |  |
| 51302 | CYP39A1 | cytochrome P450, family 39, subfamily A, polypeptide 1 | - |  |
| 57716 | PRX | periaxin | O |  |
| 284161 | GDPD1 | glycerophosphodiester phosphodiesterase domain containing 1 | - |  |

* These eleven genes considered as potential interactors of ORM2 were obtained from five interactome databases: Biological General Repository for Interaction Datasets (BioGRID), the Human Protein Reference Database (HPRD), Human Transcriptional Regulation Interactions database (HTRIdb), IntAct molecular interaction database (IntAct), and Search Tool for the Retrieval of Interacting Genes/Proteins (STRING).
